# Role of Molecular, Crystal, and Surface Chemistry in Directing the Crystallization of Entacapone Polymorphs on the Au(111) Template Surface ⊥

**DOI:** 10.1021/acs.cgd.3c00294

**Published:** 2023-05-01

**Authors:** Cai Y. Ma, Dawn Geatches, Ya-Wen Hsiao, Ana Kwokal, Kevin J. Roberts

**Affiliations:** † Centre for the Digital Design of Drug Products, School of Chemical and Process Engineering, University of Leeds, Leeds LS2 9JT, U.K.; ‡ Science and Technology Facilities Council, Daresbury Laboratory, Sci-Tech Daresbury, Warrington WA4 4AD, U.K.; § PLIVA Croatia Ltd., R&D, P. B. Filipovica 25, Zagreb 10000, Croatia

## Abstract

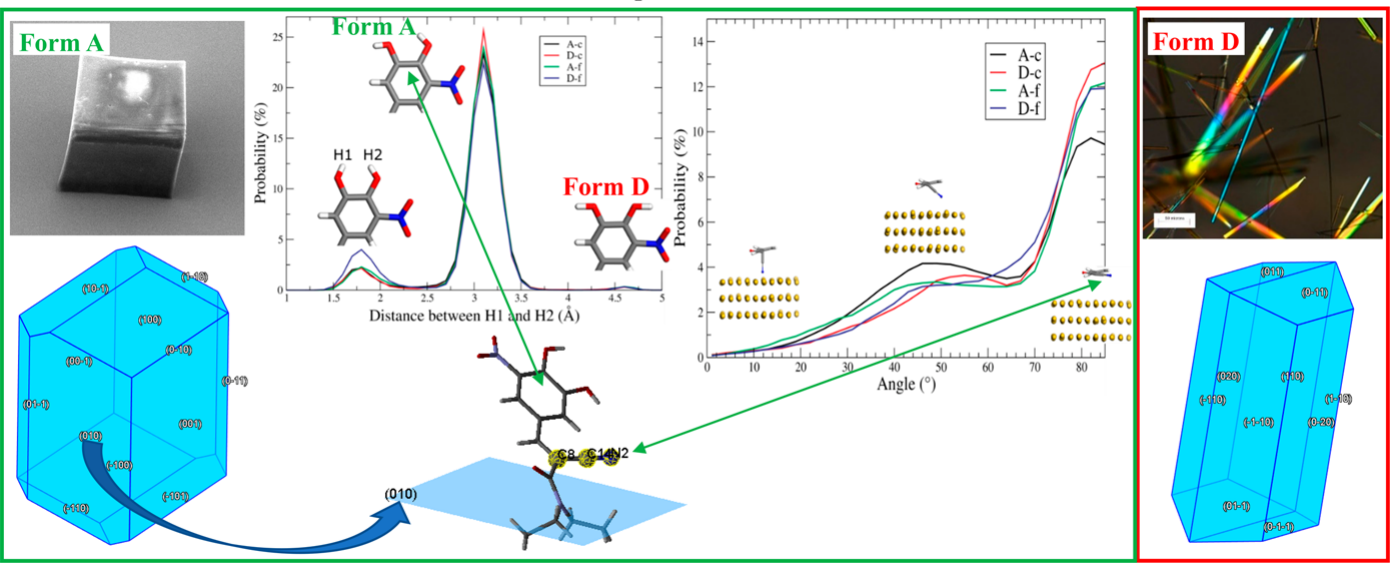

The pharmaceutical compound entacapone ((*E*)-2-cyano-3-(3,4-dihydroxy-5-nitrophenyl)-*N*,*N*-diethylprop-2-enamide) is important in the treatment of Parkinson’s disease, exhibiting interesting polymorphic behavior upon crystallization from solution. It consistently produces its stable form A with a uniform crystal size distribution on the surface of an Au(111) template while concomitantly forming its metastable form D within the same bulk solution. Molecular modeling using empirical atomistic force-fields reveals more complex molecular and intermolecular structures for form D compared to form A, with the crystal chemistry of both polymorphs being dominated by van der Waals and π–π stacking interactions with lower contributions (ca. 20%) from hydrogen bonding and electrostatic interactions. Comparative lattice energies and convergence for the polymorphs are consistent with the observed concomitant polymorphic behavior. Synthon characterization reveals an elongated needle-like morphology for form D crystals in contrast to the more equant form A crystals with the surface chemistry of the latter exposing the molecules’ cyano groups on its {010} and {011} habit faces. Density functional theory modeling of surface adsorption reveals preferential interactions between Au and the synthon G_A_ interactions of form A on the Au surface. Molecular dynamics modeling of the entacapone/gold interface reveals the entacapone molecular structure within the first adsorbed layer to show nearly identical interaction distances, for both the molecules within form A or D with respect to the Au surface, while in the second and third layers when entacapone molecule–molecule interactions overtake the interactions between those of molecule–Au, the intermolecular structures are found to be closer to the form A structure than form D. In these layers, synthon G_A_ (form A) could be reproduced with just two small azimuthal rotations (5° and 15°) whereas the closest alignment to a form D synthon requires larger azimuthal rotations (15° and 40°). The cyano functional group interactions with the Au template dominate interfacial interactions with these groups being aligned parallel to the Au surface and with nearest neighbor distances to Au atoms more closely matching those in form A than form D. The overall polymorph direction pathway thus encompasses consideration of molecular, crystal, and surface chemistry factors.

## Abstract 2

Molecular, crystal and surface chemistry examinations revealed that the adsorption and orientation of entacapone form A to the Au surface is both energetically and structurally more favorable than form D, providing a polymorph direction mechanism for nucleating form A on an Au template.

## 1 Introduction

Crystal nucleation is an important step in industrial crystallization processes and one that is often promoted by the presence of active surface sites.^1^ Nucleation is often heterogeneous, and the detailed mechanistic behavior, at the molecular scale, of such behavior is poorly understood. Many pharmaceutical active ingredients exhibit poor crystallizability, and this can, in turn, result in the generation of small particle sizes reflecting the need to generate the high solution supersaturation needed to initiate nucleation. The addition of seed crystals can enable crystallization at lower supersaturations, but the quality and uniformity of seed crystals can be quite difficult at times to control.^2^ The introduction of a well-defined and characterized structural surface template provides a potential alternative technology for controllable seeding. It has been found that in the presence of structurally ordered templates, the nucleation process can be manipulated through their ability to enable the formation of specific intermolecular binding interactions (synthons) between the active sites on the template surface and the solvated crystallizing material in the solution phase.^1,3−5^ This approach has resulted in the formation of crystalline materials with improved product properties such as shape, crystallinity, and polymorphic form.^2,6,7^ It is well-known that noble metals can possess energetically high surface area planes with the capability to readily adsorb organic molecules and thus, through this, produce novel templates with well-ordered and controlled “seed” surfaces.^1,3,4,8−10^ Several types of such interfacial templates have previously been demonstrated to have this effect, notably, single crystal surfaces,^1,8^ self-assembled layers,^3,4,9^ and Langmuir–Blodgett films,^10^ all of which can be generally considered to be ordered molecular surface systems.

A prerequisite property for such a templating surface lies in its intrinsic ability to facilitate the specific adsorption and assembly of the molecules needed to be crystallized. Such molecules could also contain functional groups that mimic those of the crystallizing species. Previous studies have also highlighted that crystallization behavior can be strongly influenced by the material used in the construction of crystallization vessels,^11,12^ implying such surfaces can also act as sites for heterogeneous nucleation. Indeed, it is a common anecdotal observation in industrial crystallization practice that the first crystallization process undertaken within a freshly cleaned reactor can be quite different in nature in comparison with subsequent crystallizations of the same system (see, e.g., ref (6)) within the same vessel. It has also been shown that the nature of the solid form (polymorph, morphology etc.) could be changed by modifying the structure and interfacial properties of the templates, e.g., using single crystals of metals such as gold,^13^ organic crystals,^1,8,14^ polymers,^15−17^ Langmuir monolayers,^5^ and surface-assembled monolayers.^18−20^

A well-characterized example of templating has been provided by the pharmaceutical compound entacapone^2,6,7,21^ which is important in the treatment of Parkinson’s disease,^22,23^ and one that also meets the “Lipinski rule of 5”^24^ criteria for its representative molecular and crystallographic parameters^25^ within the pharmaceutical drug subset.^26^ Entacapone has two well-characterized polymorphic forms (A and D), each of which have distinctly different crystal structures, external morphologies and crystallization behavior.^2^ Bommaka et al.^27^ has characterized the crystal structures, phase transformations, stability, equilibrium solubility, dissolution, and permeability properties of a range of entacapone polymorphic forms.

## 2 Entacapone Crystallization in the Presence of a Solution-Treated Au(111) Template

**Figure 1:**
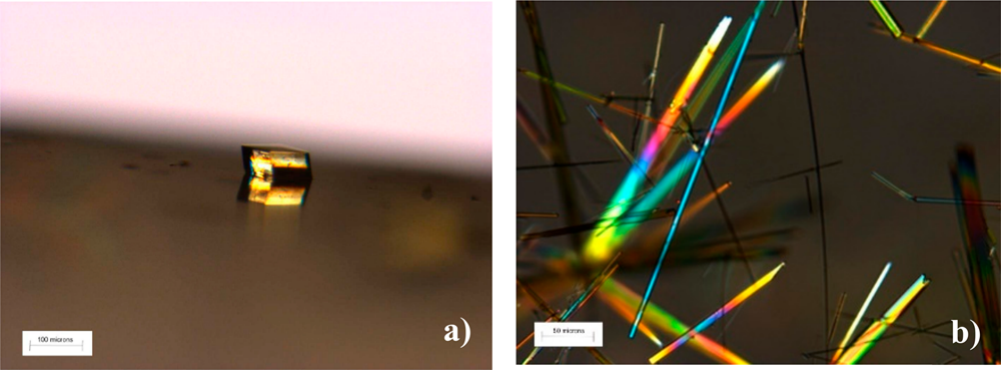
Optical micrographs of entacapone crystals (a) form A on Au(111) produced following quiescent crystallization from supersaturated acetone/water solution of entacapone and (b) fibrous crystals of form D crystallized concomitantly in the bulk solution at the bottom of a beaker.

**Figure 2:**
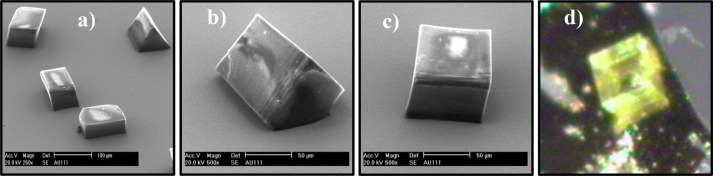
(a–c) SEM of entacapone crystals form A on Au(111) surface produced following quiescent crystallization from supersaturated acetone/water solution of entacapone; (d) optical micrograph of entacapone form A crystal taken off the surface and mounted at capillary for the purpose of plane indexing. Note that the black background area is a layer of mica.

Previous work on this system has been carried out by Kwokal et al.^2,7^ See Section S1 in the Supporting Information for experimental details. The work found that quiescent crystallization of entacapone in the presence of a surface template on Au(111) in an acetone/aqueous solution resulted in the formation of prismatic crystals of form A exclusively on the Au(111) surface (Figure 1a and Figure 2a–d), which remained attached to the surface after taking the template out of solution. In the same solution, fibrous crystals of form D had crystallized concomitantly at the bottom of a beaker within the bulk solution (Figure 1b). Examination of SEM images of the template surface revealed that the entacapone crystals attached to the template surface exhibited well-defined and orientated single crystals consistent with their epitaxial growth (Figure 2). An Au(111) surface used as a template was prepared by sputtering gold on a freshly cleaned mica surface with the surface of Au(111) being almost atomically smooth. In order to index the associated interfacial crystal plane by XRD, form A crystal was removed from the template surface by peeling off the top layer of the mica and with it the template with its adhered entacapone crystals. XRD analysis of the preferred orientation of the crystals (Figure 2d) revealed that the {010} and in some cases {011} surfaces of form A were attached to the Au(111) surface.^7^

**Figure 3:**
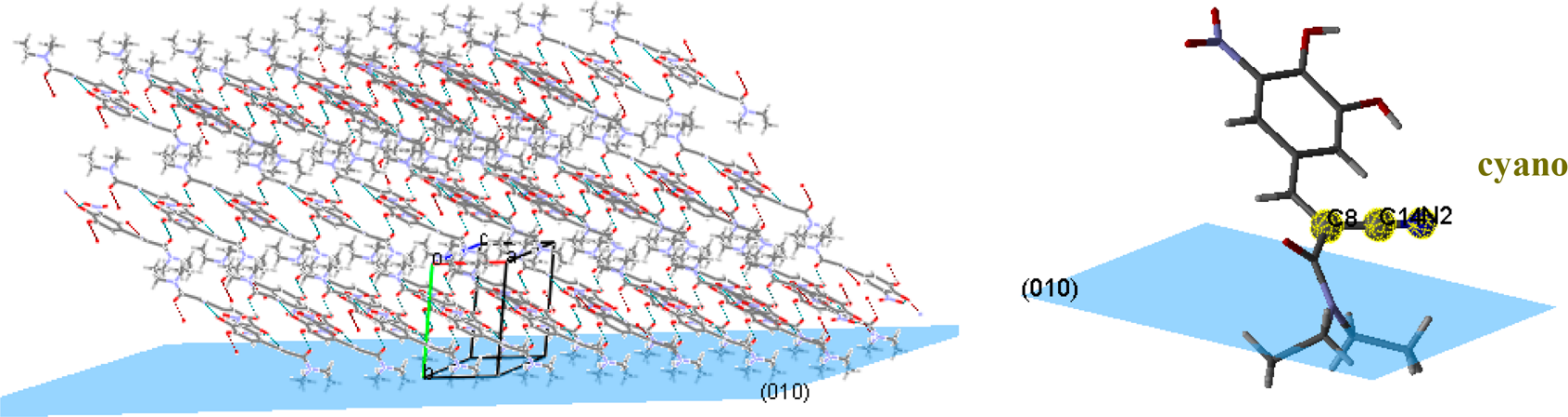
Intermolecular packing of entacapone form A in respect to the plane {010} crystal habit plane. The blue shadowed area represents the location of the {010} crystal plane.

The intermolecular packing of entacapone form A together with the crystal plane {010} (cleaved and colored in blue) (Figure 3) revealed that the surface-terminated functional groups cleaved at the {010} and {011} planes were amino groups, with the cyano group laid almost parallel to and having a ∼60° angle with the entacapone {010} and {011} planes, respectively. The Au–CN bond is well-known to be the strongest bonding functionality to gold after thiols and mercaptan groups.^2,21^ Strong Au–CN bonding has also been supported by density-functional studies^28^ in the adsorption of isocyanides on Au{111} surfaces with the CN group serving as an “alligator clip” to connect a molecule to the metallic surface. These studies revealed that adsorption was possible at all (hollow or atom) Au(111) surface sites, with hollow sites preferred with the adsorption energies for both HNC and CH_3_NC molecules being calculated to be 0.2 and 1 eV,^21^ respectively.

Studies of the orientation of molecules with a similar functionality, such as benzonitrile on Au, revealed that the molecules initially adopt a flat orientation (via π-bonding) but that these can reorient from the flat to a vertical or tilted (σ-N-bonded) state depending on the surface potential.^29,30^

Surface enhanced Raman spectroscopy studies of entacapone on Au(100) at an open circuit potential confirmed the adsorption of entacapone on gold. However, detailed analysis of the associated Raman spectra revealed that the adsorbed layer structure did not fully resemble either that of form A or D, albeit the CN stretching vibrations were found to have the same ν-shift for both the A and D forms of ∼ 2270 cm^–1^.^2^

Cyclic voltammetry and impedance studies of entacapone on Au(100) at zero charge (∼0–200 mV vs Ag/AgCl) in acetone/aqueous solutions revealed a strong adsorption of entacapone; with capacity as low as 0.24 μF cm^–1^ after 8 h, the latter indicating both a homogeneous and relatively thick adsorbed layer.^2^

Overall, the work to date confirms that entacapone readily adsorbs on Au surfaces and can be assumed to provide a nucleation template for directing the epitaxial surface crystallization of the form A of entacapone. In particular, the work suggests that the cyano functional group of entacapone is orientated parallel to the Au template surface and interacts through the formation of strong C≡N···Au bonding. However, the mechanism by which entacapone crystallizes as form A and not form D on the template surface is still not that well understood.

In this paper, the latter aspect has been examined through further study which draws upon a detailed examination of the molecular, crystal and surface chemistry of both the A and D polymorphic forms using empirical force field modeling of intermolecular interactions, together with density functional theory (DFT) and molecular dynamics (MD) studies of entacapone surface binding and subsequent the adsorption at the solution-treated Au(111) surface, respectively. The overall aim of the study has been to provide an insight as to how, at the molecular-scale level, the solution-treated template induces and directs the pathway to form A rather than form D through the nucleation process.

## 3 Materials and Methods

### 3.1 Experimental Details

#### 3.1.1 Materials

Entacapone, (*E*)-2-cyano-*N*,*N*-diethyl-3-(3,4-dihydroxy-5-nitrophenyl)propenamide, with a purity of approximately 99%, was provided courtesy of PLIVA Croatia. The crystallization solvent was acetone. Entacapone form A crystallizes in a triclinic system crystal.^31^ An Au(111) surface film was sputtered on a freshly cleaned mica surface and used as the substrate for the surface template.^2^ The crystal structure of form D was solved as part of this study.

#### 3.1.2 Crystal Structure Determination

Data collection of entacapone form D on a single crystal was carried out at Pliva Pharmaceuticals in Croatia at room temperature (297 K), with Cu Kα radiation (λ = 1.54180 Å) using an Oxford Diffraction Xcalibur diffractometer with a Sapphire CCD detector, in a *Q* range of 3.38–61.4° and omega-scan data collection method using CrysAlisPro^32^ revealing an absorption correction was done by CrysAlis RED.^32^ An absorption factor of *m* = 0.99 mm^–1^ was used. Minimum and maximum transmissions were *T*_min_ = 0.805, *T*_max_ = 0.834, respectively. There were 9317 measured reflections and 2394 independent reflections, with 1127 reflections having *I* > 2σ(*I*). The internal reflections factor was *R*_int_ = 0.069.

The crystal structure was solved and refined using SHELXS97^33^ and SHELXL97,^33^ respectively. The full refinement details, based on 2394 reflections, are given in the Supporting Information, Section S2 (Table S1).

### 3.2 Modeling Methods

**Figure 4:**
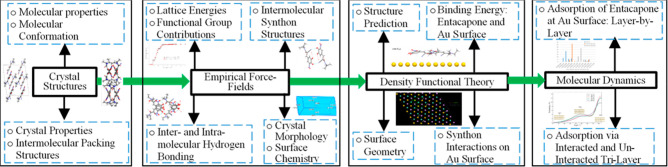
Schematic workflow structure highlighting the interconnectivity between molecular and crystal properties, synthonic structures and surface chemistry, DFT binding energies, and MD adsorption of entacapone at the Au surface, demonstrating the importance of molecular-scale understanding with combined molecular modeling, DFT and MD.

The interconnectivity between molecular and crystal properties, synthonic structures and surface chemistry, DFT binding energies and MD adsorption of entacapone at Au surface are shown diagrammatically as a workflow (Figure 4), highlighting the importance of molecular-scale understanding with combined empirical force field modeling,^34,35^ density functional theory (DFT)^36^ and molecular dynamics (MD).^37^ The definitions and purposes of the parameters calculated in this study are listed in Table S16 (Supporting Information).

#### 3.2.1 Molecular and Crystallographic Modeling

**Figure 5:**
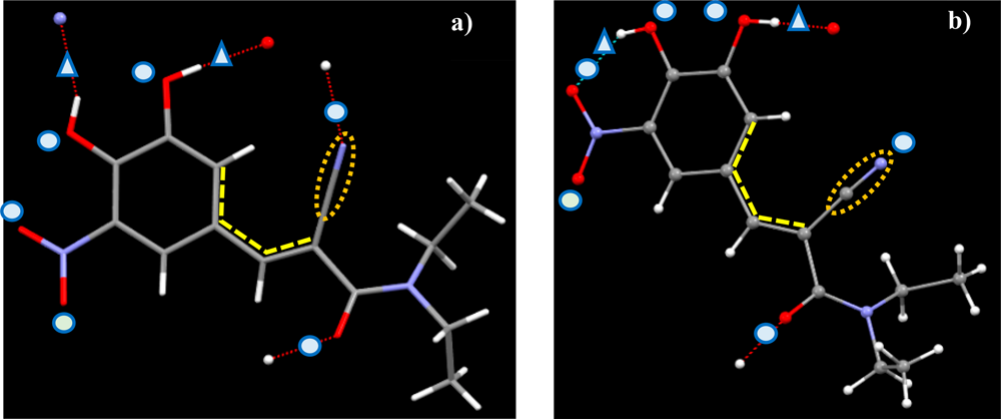
Molecular structures of entacapone (a) polymorph form A and (b) form D with their hydrogen bonding donors (triangle) and acceptors (circle). The torsion for molecular conformation analysis is indicated by yellow dashed lines and the C≡N group by a brown dotted oval.

The molecular and crystal structures of entacapone form A^31^ (ref code: OFAZUQ) with two molecules in the unit cell and one crystallographically independent molecule (conformer) in the asymmetric unit in a triclinic crystal system (space group P-1), were obtained from the Cambridge Structural Database (CSD).^38^ Molecular descriptors were calculated using the CSD Python API.^38,39^ Further analysis and refinement was carried out using Materials Studio,^40^ Conquest V1.18^41^ and Mercury V2020.2.0.^42^ A torsion (yellow dashed lines in Figure 5) was identified based on the existence of the tail part rotations and the importance of the cyano functional group for the possible interactions with an Au surface. The energy variations with the torsion angles (−180° to +180°) of forms A and D molecules were calculated using Materials Studio^40^ with the energetically ranked top two molecular structures being identified for comparison with the molecules in forms A and D (see further details in the Supporting Information, Section S3).

The intermolecular pair interaction energies for the two polymorphic structures were calculated using HABIT98^34,35^ with the Dreiding force field^43^ and MOPAC^44^ atomic charges. The lattice energy was calculated together with its convergence which was analyzed using both cumulative and discretized radial interactions (see full details in the Supporting Information, Section S5.1).

The morphologically important faces associated together with their growth layer thickness^45^ were identified and ranked by the BFDH method^46−49^ using Mercury.^39^ Dominant intermolecular interactions, identified in the lattice energy calculations, were partitioned between the intrinsic (bulk) and extrinsic (surface-terminated) synthons^50,51^ and the associated surface attachment energies calculated and, through this, the morphologies predicted (see further details in the Supporting Information, Section S5.2).

The intermolecular surface chemistry of the selected crystal habit surfaces, together with their constituent synthons, were visualized using Materials Studio and tabulated on a face (*hkl*)-specific basis (see further details in the Supporting Information, Section S5.3).

#### 3.2.2 DFT and MD Studies of Intermolecular Interactions

Dynamic atomistic modeling including DFT^36^ and MD^37^ were used to simulate the binding energies of the interactions between entacapone molecules and the Au(111) surface and the subsequent adsorption process, respectively.

DFT-based atomistic modeling (software CASTEP^52^) was used to explore the adsorption energy of both single molecules and intermolecular dimers (analogous to synthons) interacting with an Au surface. For the DFT models, the overall representative rather than the specific motifs of interaction were identified as dimers, as every possible interacting pair (dimer) within the bulk crystal structures may differ only by minor configurational changes. The entacapone single molecules and molecular pairs (synthons) were initially placed above the Au surface with their Au–N distances and C≡N–Au angles being set within the experimental parameters, following which the molecules and synthons were optimized to their local configurational minima.

MD (software NAMD^37^) was used to simulate the interactions between the Au atoms in an Au template surface and entacapone molecules adjacent to the template. The MD models comprised a series of 1, 2, and 3 layers of entacapone oriented above an Au surface. The layer-by-layer adsorption model was used to examine the consistency of form A adsorbed on the Au surface with 1, 2, and 3 layers of entacapone molecules. As the simulation results (Section 4.6) revealed that form A molecules were found to be dominant consistently in 1-layer, 2-layer, and 3-layer adsorption structures, adding further layers would be expected to produce similarly consistent adsorption structures. During the MD simulations, the 3-layer slab of gold atoms was held fixed (following its initial relaxation in the absence of adsorbed molecules) and the entacapone molecules were fully flexible. Following the simulations, the locations and orientations of entacapone molecules, and the interactions between Au and entacapone molecules, were analyzed. Further computational details for the DFT and MD modeling are described in the Supporting Information, Sections S7.1 and 7.2, respectively.

Note that the software, HABIT98, has been implemented in the CCDC’s Mercury^42^ under CSD-Particle module (VisualHabit) through the close collaboration with the CCDC. Further integration of DFT/MD open source codes in the future could create a single modeling platform for wider applications.

## 4 Results and Discussion

### 4.1 Crystal Structure of Entacapone Form D

Entacapone form D was found to crystallize in an orthorhombic crystal structure (space group *Pna*2_1_) with eight molecules in the unit cell and two crystallographically independent molecules (conformers) in the asymmetric unit; its crystallographic data are summarized in Table 1, together with the details of form A^31^ (as the triclinic system crystal, available in the Cambridge Crystallography Database (CSD) as “OFAZUQ”), and the recently published crystallographic parameters of form D (referred to as form II in ref (27)). The full crystal structural report for entacapone form D is summarized in the Supporting Information (Table S2) and is also available in the CSD (deposition number 2209890), with the atomic coordinates of forms A and D being given in the Supporting Information, Section S4 (Table S3). The crystallographic parameters of form D from this study and those recently reported^27^ were found to be very close, albeit for this study the form D’s structural parameters based upon the structure derived in this work were used. The hydrogen bond network for the two crystallographically independent molecules in form D crystal structure is given in the Supporting Information, Section S2 (Figure S1).

**Table 1:** Characteristic Molecular Descriptors and Crystallographic Structural Data for the Entacapone Polymorphs Including DFT-Optimized Results

	form A		form D		
material descriptor					form II
method	XRD	DFT	XRD	DFT	XRD
Refcode	OFAZUQ	–	2209890	–	OFAZUQ02
reference	(31)	(this study)	(this study)	(this study)	(27)
molecular weight (g/mol)	305.29	305.29	305.29	305.29	305.29
molecular volume (Å^3^)	266.78	–	264.61/266.24	–	264.84/266.22
molecular surface area (Å^2^)	285.42	–	282.18/287.55	–	282.35/287.89
crystal system	triclinic	triclinic	orthorhombic	orthorhombic	orthorhombic
space group	*P*1̅	*P*1̅	*Pna*2_1_	*Pna*2_1_	*Pna*2_1_
*Z/*Z′	2/1	–	8/2	–	8/2
*a* (Å)	7.576	7.450	15.188	14.811	15.191
*b* (Å)	9.688	10.056	25.678	25.215	25.691
*c* (Å)	9.905	9.524	7.496	7.346	7.494
α (deg)	100.17	101.11	90	90	90
β (deg)	99.61	98.01	90	90	90
γ (deg)	95.81	97.40	90	90	90
cell volume (Å^3^)	699.098	684.375	2930.5	2743.43	2924.7
packing coefficient	0.725	–	0.689	–	0.689
void space (%)	24.7	–	28.4	–	28.3
density (g/cm^3^)	1.45	–	1.38	–	1.387

### 4.2 Molecular Chemistry Analysis of Entacapone

The molecular volumes and molecular surface areas of forms A and D were found to be very similar, albeit the second crystallographically independent molecule within the asymmetric unit of form D was found to have a slightly larger volume and surface area when compared to the first one. The molecules from forms A and D both have two hydrogen bond donors and six hydrogen bond acceptors (Figure 5) together with 11 rotatable bonds. The corresponding geometrical parameters of the hydrogen bonds are listed in Table 2. The alkyl tail part (amide and alkane groups) of form A molecule roughly aligns with the aromatic ring plane, hence providing a more planar molecular structure while in form D the molecular positions have the tails at ∼180° with respect to each other, hence providing less planar overall molecular conformation. The top-ranked energy-minimized conformations for the molecular structures have ∼20° torsion angle difference compared to those from crystal structures. This suggests a conformational change of energy penalty associated with crystallization albeit only a small one with a < 1.8 kcal/mol difference in the conformational energies of all calculated conformations, indicating overall that the crystal packing considerations would be expected to be dominant in the crystal structural energy balance. This is consistent with the behavior of many small molecule pharmaceuticals.^25^ The torsion angles involving CN group for the form A molecule and two form D molecules have differences about 40°, indicating similar exposure and availability of CN group for all three molecular conformers. Further details can be found in the Supporting Information, Section S3.

**Table 2:**
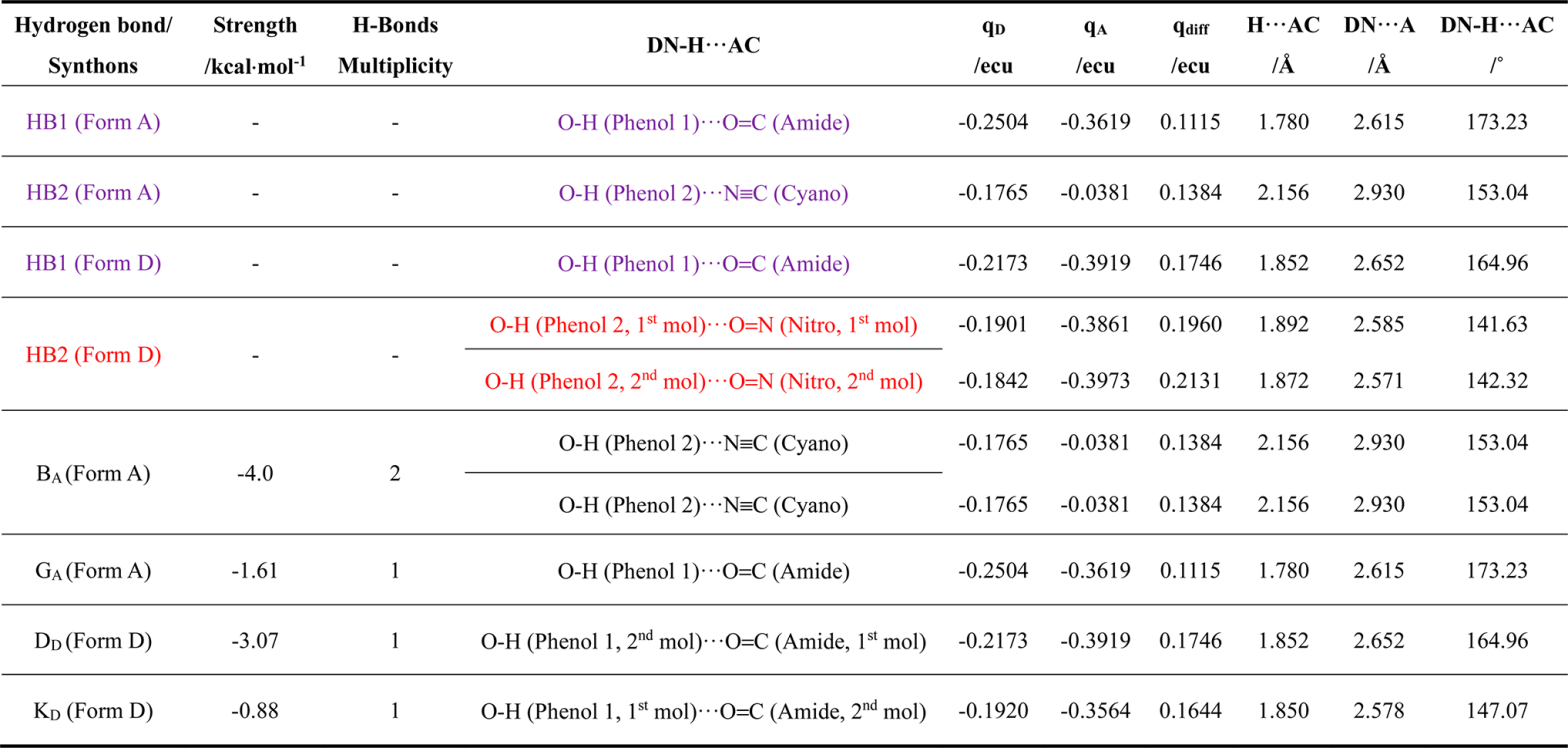
Detailed Analysis at the Atomic Level of the Constituent H-Bonding Interactions Involved in the Hydrogen Bonds (HBs) for Forms A and D, and Four H-Bonding Synthons (Supporting Information, Tables S7 and S8) Highlighting the Geometrical Details of the Contribution Donor (DN) and Acceptor (AC) Sites together with Their Respective Polarizability^a^

a The hydrogen atom in the phenol group is in the same position as the configuration used to calculate lattice energy. For clarity, O= and N≡ denote the double bonded oxygen and triple bonded nitrogen to a carbon with the inter- and intra-molecular HBs being colored as purple and red, respectively.

### 4.3 Crystal Chemistry Analysis of Entacapone

#### 4.3.1 Comparative Intermolecular Packing

The unit cell size of form D along the *c*-axis is about one-third of the size of its length along the *b*-axis and half of the length along the *a*-axis, forming an elongated plate-like unit cell. The structure of form D includes one intramolecular and one intermolecular hydrogen bond, whereas form A has two intermolecular hydrogen bonds. As listed in Table 2, form A has two hydrogen bonds with two phenol groups binding to amide and cyano groups, respectively, while form D has a similar O–H(Phenol 1)···O(amide) hydrogen bond and an intramolecular hydrogen bond: O–H(Phenol 2)···O(Nitro). This indicates that the CN group in the form D conformer presents less potential for hydrogen bonding interaction in its crystal structure, hence also less probability of forming a binding interaction with Au surface. The lower symmetry of form A (*Z* = 2, *Z*′ = 1) gives rise to a much simpler crystal structure and intermolecular packing than form D (*Z* = 8, *Z*′ = 2). As a result, form A is a more close-packed crystal structure with a concomitantly lower void volume percentage compared to form D, leading overall to a higher packing coefficient and crystal density, consistent overall with its higher relative stability.

**Figure 6:**
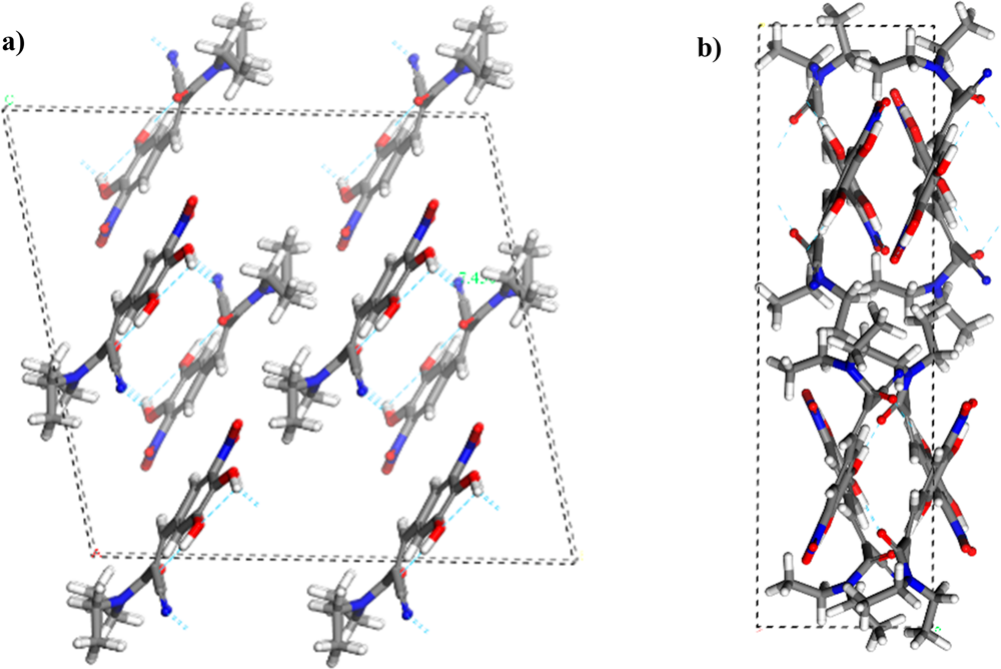
Bulk crystal of entacapone: (a) 2 × 2 × 2 supercell of form A; (b) unit cell of form D (N = blue; C = gray; O = red; H = white).

The entacapone molecules in form A were found to align themselves parallel to the crystallographic lattice plane (0–11), and in form D rows of parallel pairs of molecules align themselves alternately along the (0–11) and (011) lattice planes, forming an interlocking criss-cross pattern (Figure 6).

#### 4.3.2 Lattice Energies, Their Convergence, and Functional Group Contributions

**Figure 7:**
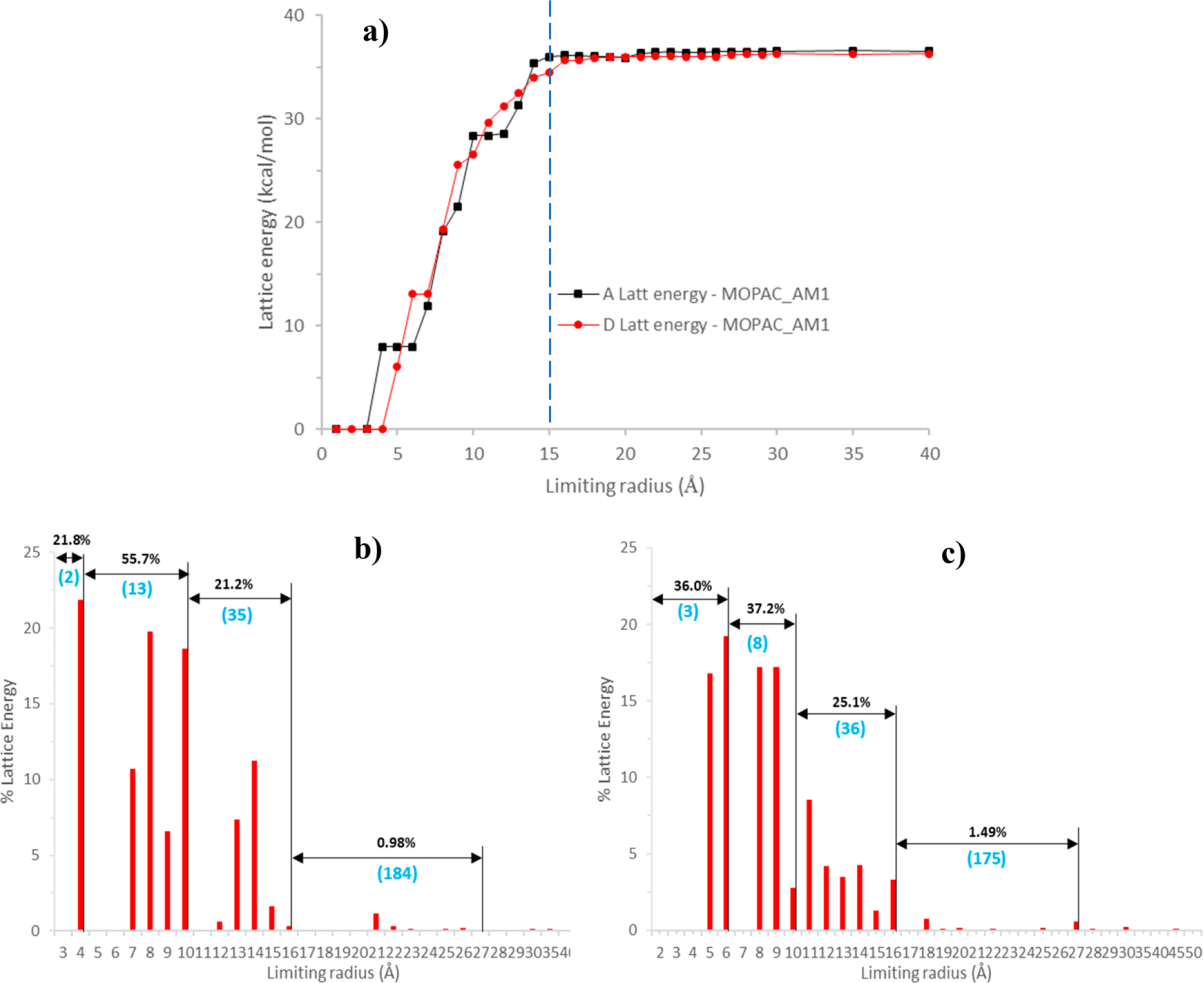
Convergence of the intermolecular summation associated with the determination of (a) the lattice energy and (b, c) radial discretized distribution plots showing the % contribution to the lattice energy as a function of intermolecular summation distance for (b) form A and (c) form D.

Figure 7 and Table 3 summarize modeling data regarding the convergence of the lattice energies for both forms A and D, providing both radial and discretized intermolecular energy distribution plots that highlight the % contribution to the lattice energy as a function of intermolecular summation distance. There is a reduction in the percentage contribution with an increasing radial intermolecular distance highlighting the short-range of the intermolecular interactions and significance of nearest neighbor synthons in terms of the crystal lattice stabilization. The data reveal that the two polymorphs have quite similar energetic pathways in terms of their molecular assembly at nucleation which is consistent with their concomitant polymorphic behavior,^2^ in contrast to other molecular compounds such as *p*-aminobenzoic acid,^53,54^l-glutamic acid,^55^ and ritonavir.^56,57^

**Table 3:** Percentage of the Lattice Energy Added and the Number of Molecules with Increasing Intermolecular Summation Distance Covering the Various Coordination Shells

		number of molecules		% lattice energy	
coordination shells	distance range (Å)	form A	form D	form A	form D
1	0–6	2	3	21.8	36.0
2	6–10	13	8	55.7	37.2
3	10–16	35	36	21.2	25.1
4	16–27	184	175	0.98	1.49
Total	0–27	234	222	99.68	99.79

Examination of the molecular polarizability reveals no significant differences between the molecules in the two polymorphic forms except for the oxygen (O2) in the phenol group (away from the nitro group) and O4, O5 in the nitro group, and carbons (C3, C5) in the aromatic ring group (see a full list of the calculated atomic charges in the Supporting Information, Section S4 (Table S4)).

Examination of the respective contributions from the seven functional groups within the entacapone molecule, phenol (×2), nitro, aromatic ring, alkene, cyano, amide, and alkane (×2), reveals that for both forms A and D, the aliphatic (including one alkene and two alkane) and aromatic ring groups make contributions of 61.34% and 57.18% to the corresponding lattice energies, respectively. This is consistent with the dominance of dispersive interactions in the crystal lattice. It should also be noted that the nitro, amide, cyano and two phenol groups have hydrogen-bond acceptors and/or donors and may be involved in hydrogen-bond interactions (see further information in the Supporting Information, Section S5 (Table S6)).

#### 4.3.3 Intermolecular Synthon Analysis

**Figure 8:**
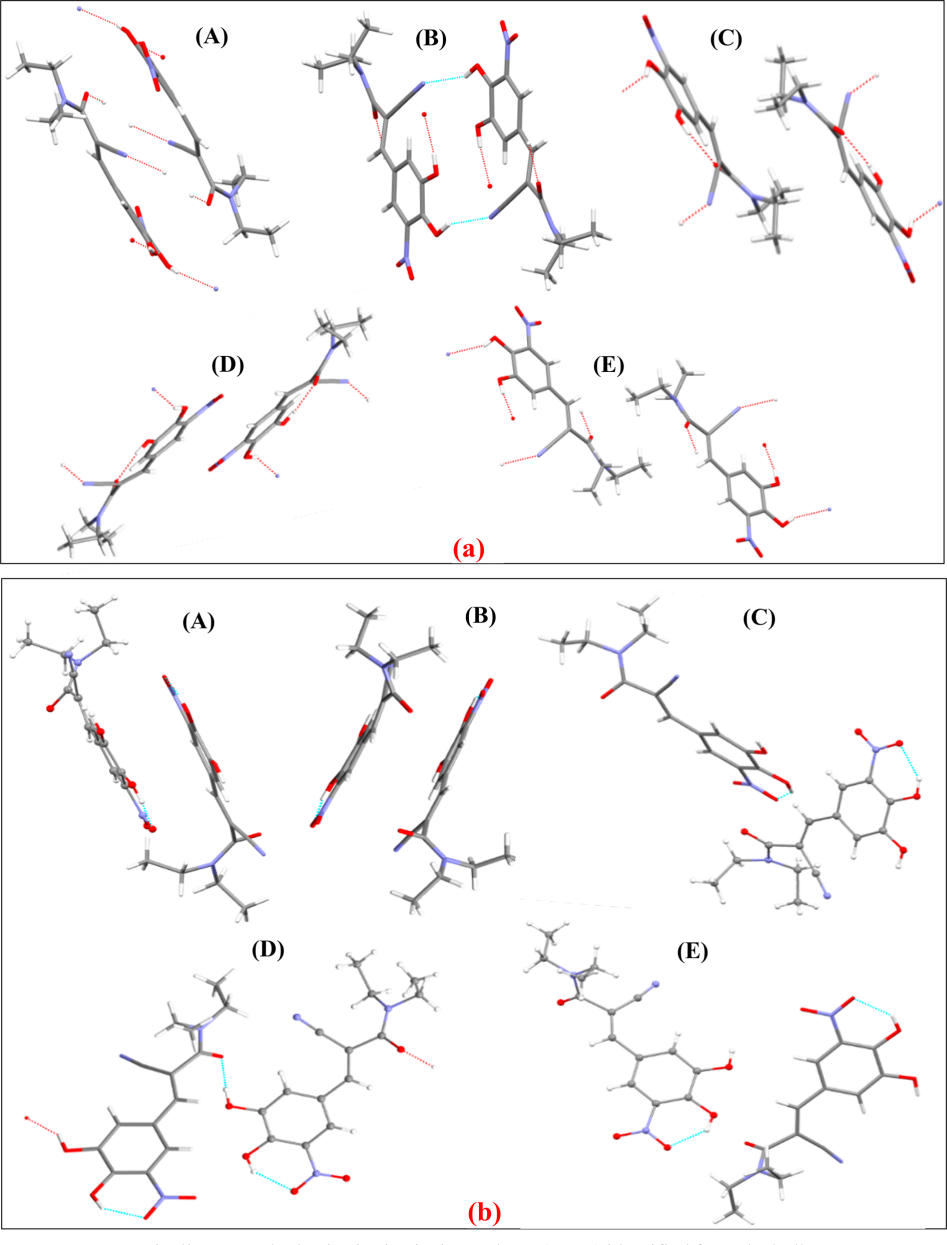
Energetically top-ranked pairwise intrinsic synthons (A – E) identified from the bulk structure of entacapone (a) form A and (b) form D (1st molecule (stick); 2nd molecule (ball and stick)).

Figure 8 shows the intermolecular structures for the energetically ranked top 5 intrinsic (bulk) synthons (A–E) in the form A and D structures.

In the form A structure, the strongest synthon A_A_ results from a strong π–π stacking interaction between the aromatic groups which is ca. 4 kcal/mol larger in energy compared to synthon B_A_. Synthons B_A_ and G_A_ involve hydrogen-bonded structures (blue dotted lines in Figure 8a(B_A_) and also in the Supporting Information, Section S5 (Figure S8a)(G_A_)), while the remaining 8 synthons (C_A_, D_A_, E_A_, F_A_, H_A_, I_A_, J_A_, K_A_) are all dominated by van der Waals interactions.

The two top-ranked synthons (A_D_ and B_D_) for form D result from a π–π stacking interaction between the aromatic groups with their van der Waals interactions contributing the most to the synthon energy (see in the Supporting Information, Section S5 (Table S10)). Synthon G_D_ also has a π–π stacking structure. Synthon D_D_ and synthon K_D_ form hydrogen-bonds (blue dotted lines in Figure 8b(D_D_) and also in the Supporting Information, Section S5 (Figure S8b)(K_D_)). The other 6 synthons (C_D_, E_D_, F_D_, H_D_, I_D_, J_D_) are dominated by van der Waals interactions.

In comparative terms, it was found that all form A synthons (Figure S8a) involved comparatively parallel inter-molecular interactions with respect to each other with sliding and/or translating operations of the molecules but without any significant rotation, consistent with this form’s low symmetry structure and also its more planar molecular structure. In contrast, the higher symmetry and more complex synthonic structure of form D, shown in Figure S8b, indicates that rotating, sliding, and translating operations of the two molecules lead to the formation of its constituent synthons. Full details of the structures and properties of the top ranked 11 synthons of forms A and D are given in the Supporting Information, Section S5 (Figure S8 and Tables S7–S10).

#### 4.3.4 Inter- and Intra-molecular Hydrogen Bonding Analysis

The detailed breakdown of the constituent hydrogen bonds associated with synthons B_A_, G_A_ of form A and D_D_, K_D_ of form D is given in Table 2. Analysis of form A reveals it has two hydrogen-bonded synthons: B_A_ comprising two identical hydrogen bonds: O–H (phenol)···N≡C (cyano), and G_A_ comprising one: O–H (phenol)···O=C (amide) hydrogen-bond. Form D also has two hydrogen-bonded synthons: D_D_ with an O–H (phenol, second molecule)···O=C (amide, first molecule) hydrogen bond and K_D_ which has one O–H (phenol, first molecule)···O=C (amide, second molecule) hydrogen bond. As shown in Table 2, form A has two identical OH−N interactions for synthon B_A_ and one OH–O interaction for G_A_, while form D has one OH–O interaction each for both synthons (D_D_ and F_D_) but with the hydrogen bond donors and acceptors from different molecules in the crystal’s asymmetric unit.

Overall, the more close-packed structure, with a lower void percentage and higher density of form A compared to the metastable form D is reflected also in the analysis of the top hydrogen bonding synthon structures. In this, those in form A are stronger ((−4.0 kcal/mol) when compared to form D (−3.07 kcal/mol). On a per bond basis, the hydrogen bonds are stronger in form D (−3.07 kcal/mol) than in the form A ((−2.0 kcal/mol) as evidenced by the latter’s hydrogen/acceptor distance (1.852 Å) being ca. 14% shorter than form A (2.156 Å), which may be a reflection of its higher symmetry, criss-cross packing structure, and also less planar molecular structure.

### 4.4 Surface Chemistry Analysis of Entacapone

**Figure 9:**
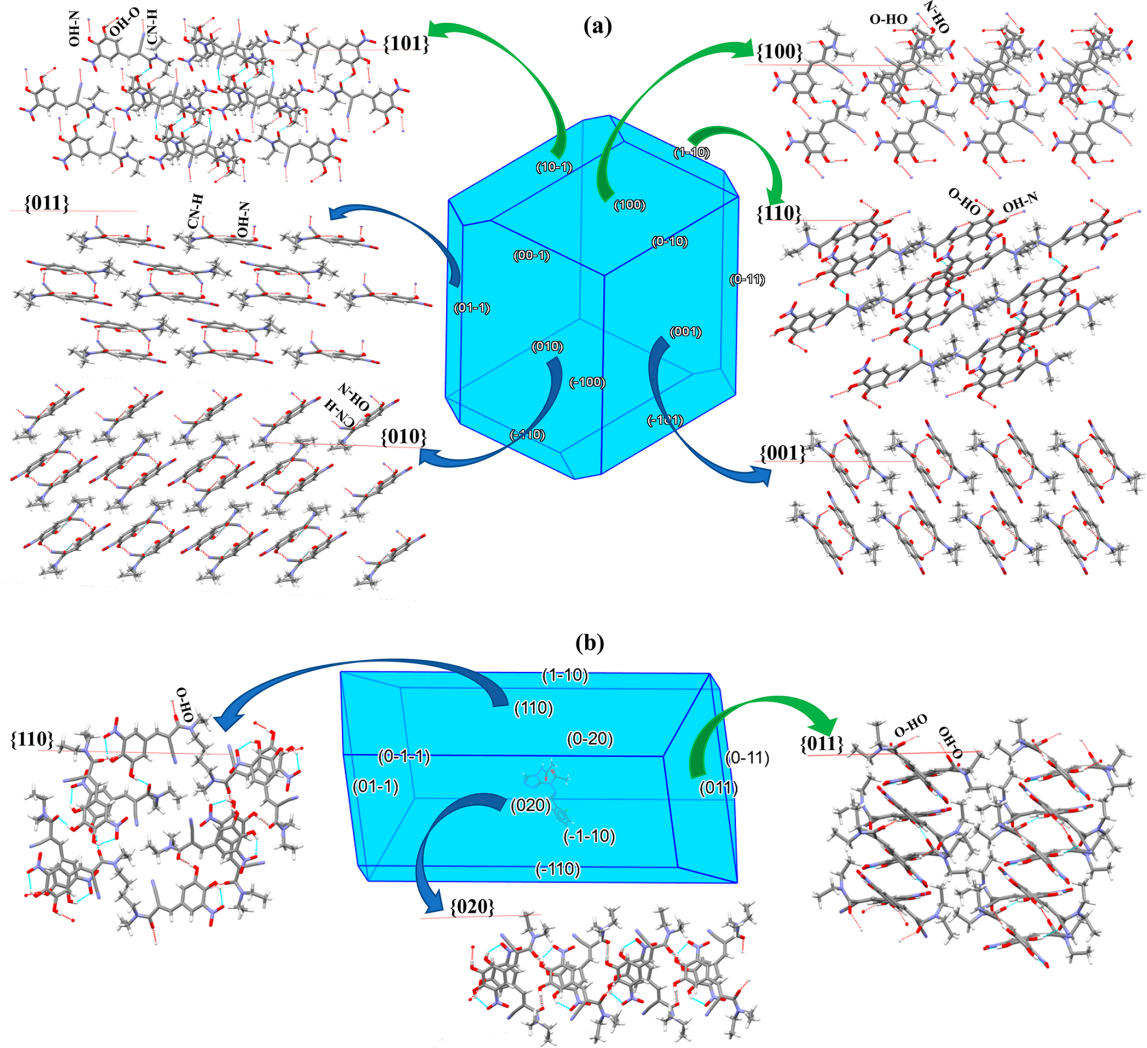
Predicted crystal morphology of (a) form A and (b) form D, highlighting the expected surface chemistry of the morphologically most important crystal habit faces, in particular, the availability of CN-H and OH-O hydrogen bonds with the former for the possible interactions between the CN group and the Au template.

The results of the 3D morphological simulations together with the associated surface chemistry of the dominant crystal habit faces for the two polymorphs is given in Figure 9. A comparative analysis of the attachment energies and the associated percentages of surface saturation levels present in their predicted crystal habits, revealed the {101}, {110}, and {100} capping faces of form A (Figure 9(a)) are only about 4 kcal/mol larger and 10.8% higher than the {001}, {010} and {011} side faces. This would suggest that the differences between the expected crystal growth rates for all the crystal faces might not be high, as would be consistent with an equant habit.^2,6,7,27^ For form D, the capping face {011} (Figure 9b) has an attachment energy of −16.46 kcal/mol and saturation level of 55% which are much larger than −7.6 kcal/mol and much lower than 79% of the side face {020}, respectively, hence consistent with a long plate-like or thin fibrous crystal shape as observed for form D.^7,27^

The surface chemistry analysis reveals that the particularly interesting crystal faces {010} and {011} of form A which bond to the Au template expose surface terminated CN and OH functional groups which are thus available for the formation of synthons C_A_ and G_A_, while only the OH functional group is exposed on the habit faces of form D. The CN groups exposed on the {010} and {011} faces (Figure 9(a)) subtend angles of ∼90° (parallel) and ∼60° with respect to the surfaces, respectively, clearly indicating the potential for their exposed CN groups to be available for interaction with the Au template surface. In contrast, the CN groups on the {110}, {011} and {020} faces in form D were found to be either shielded by this form’s less planar molecular structure or to be orientated normal to the {110} face (Figure 9(b)), reducing their availability for interaction with the Au template surface.

Further detailed analysis of surface chemistry for both forms A and D are given in the Supporting Information, Section S6 (Tables S11–S14).

### 4.5 Binding Energies of Entacapone with the Au(111) Surface

#### 4.5.1 Structure Prediction versus Crystallographic Data

The results of the DFT simulations of the crystal structures of forms A and D are summarized in Table 1, columns 3 and 5, with the predicted lattice parameters being found to vary by between 1 and 4% from those available in the CSD (form A) and found experimentally (form D) in this study. These results are consistent with the widely accepted benchmark for the simulation parameters (see further details in the Supporting Information, Section S7.1).

The lattice energies calculated using DFT were found to be −52.24 kcal/mol for form A, and −44.87 kcal/mol for form D (see in the Supporting Information, Section S5.2 (Table S5)). These calculations did not include the molecular vibrational free energy due to its computationally intensive requirements and relatively small contribution made to the overall lattice energy.^58^ Exploring the reasons for the differences between the results obtained using an empirical force field and those using the ab initio DFT method are outside the scope of this study but suffice it to say that, for the purposes of benchmarking results using different methods, they are comparable within the limitations of their respective methods and parameters.

The calculated lattice energies for both forms using empirical and DFT methods were found to be broadly consistent, both revealing form A to have a larger value than form D. Both forms were found to be dominated by van der Waals interactions contributing about 80% to the overall energy. However, form A has a higher hydrogen-bond contribution to its lattice energy than form D, and form D possesses a greater electrostatic contribution than form A. Full details can be found in the Supporting Information, Section S5.2 (Table S5).

#### 4.5.2 Binding Energy Calculations

**Figure 10:**
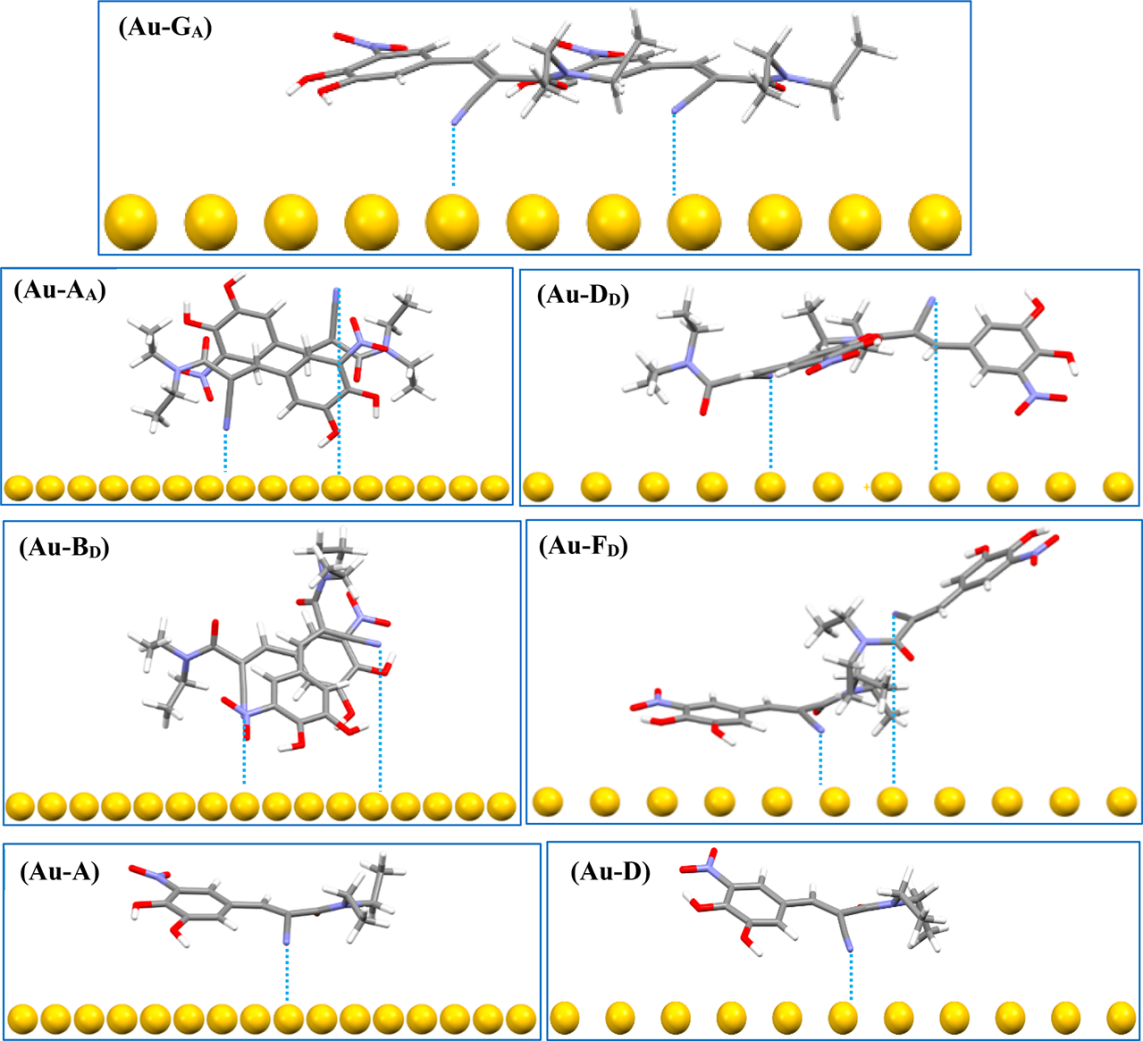
Interaction configurations of entacapone synthons and conformers of forms A and D on Au(111) surface after optimization. The dotted lines indicate the possible Au–CN interactions/bonds.

Figure 10 shows the interaction configurations of entacapone molecular conformers and inter-molecular synthons of forms A and D on the Au(111) surface after optimization with the possible Au···CN bonds highlighted by dotted lines. Comparing the binding energies (Table 4 and Table S15) of the monomers to the Au surface (Au-A and Au-D) and synthonic dimers (i.e., DFT-synthons as given in the Supporting Information, Section S7.3 (Figure S13 and Table S15)) and ranking them in the order of decreasing strength give
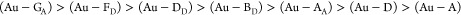


**Table 4:** DFT-Calculated Binding Energies of Entacapone Synthons and Conformers of Forms A and D on the Au(111) Surface after Optimization^a^

		C≡N–Au distance (Å)		C≡N–Au angle (deg)		comment: distance/orientation	
interaction configurations	energy (kcal/mol)	CN in 1st molecule	CN in 2nd molecule	CN in 1st molecule	CN in 2nd molecule	CN in 1st molecule	CN in 2nd molecule
Au–G_A_	–44.05	2.912	3.481	61	67	short/parallel	short/parallel
Au–A_A_	–21.29	3.264	10.143	37	168	short/vertical	long/vertical
Au–D_D_	–29.74	4.864	8.182	97	126	short/parallel	long/parallel
Au–B_D_	–26.99	4.164	7.522	12	78	short/vertical	long/parallel
Au–F_D_	–39.20	3.244	8.397	83	86	short/parallel	long/parallel
Au–A	–16.65	3.074		60		short/parallel	
Au–D	–20.97	2.978		62		short/parallel	

a The labels match those used in Figure 10. Note that the angle between CN and the Au surface is defined as 0° (CN perpendicular to Au surface) and 90° (CN parallel to Au surface).

Analysis of the ranking data reveals that it is energetically favorable for synthons to adsorb to the Au surface where their binding energy becomes stronger through a combination of shorter C≡N–Au distances, and optimized van der Waals interactions via maximum planar orientation of the projected molecule onto the template surface, for example, a comparison of the binding energies of Au–G_A_ (−44.05 kcal/mol) with its two C≡N–Au bonds, to Au–A_A_ (−21.29 kcal/mol) with larger C≡N–Au distances and edge-on orientation to the Au surface. It was also found that the C≡N–Au angle for Au–G_A_ (Table 4) can be achieved by rotating less than 30° or 10° from the CN orientation of faces {010} or {011} and has the shortest C≡N–Au interaction distances of all synthons studied. The second feature of the binding energy ranking is that, while the energy released by a single molecule favors the binding of the form D conformer rather than that for form A, the situation is reversed when binding the intermolecular synthons resulting from the directed assembly of the crystallographic structure of the two polymorphs. Indeed, a comparison between the binding energies of monomers and the dimeric synthons reveals the respective binding energies to be roughly comparable, i.e. ((Au–A: −16.65 kcal/mol) and (Au–D: −20.97 kcal/mol)), for monomer binding to the Au surface compared to those for the synthonic dimers (G_A_ = −18.46 kcal/mol, and D_D_ = −16.83 kcal/mol). In respect to this, the planar molecular structure of form A might help to form an adsorption layer of form A on the Au surface through strong double C≡N–Au interactions such as (Au–G_A_).

#### 4.5.3 Au(111) Surface Geometry and Synthons

**Figure 11:**
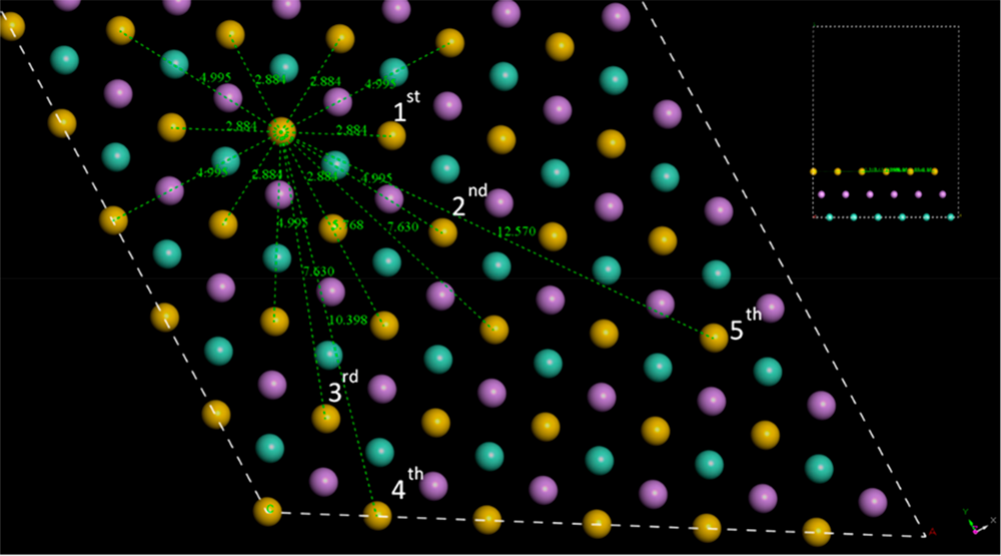
Projected view of Au(111) surface as would be accessible to entacapone molecules. Inset: side view showing different layers in ABC packing of the face-centered cubic packing of the slab. Note that distances are in Å, and the *N*th nearest neighbors in the same layer are labeled as 1st to 5th.

Mindful that the Au surface is templating the formation of form A crystals, then it is reasonable to seek a connection between geometrical features of the template surface and the form A crystallographic structure. The energy ranking above (Section 4.5.2) clearly favors the G_A_ synthon adsorption on the Au(111) surface, and a “bird’s eye” view of this surface (Figure 11) shows that the distances between nearest neighbor Au atoms in the Au surface (Table 5) more closely match the distances between CN bonds oriented in the same cis- direction in form A (i.e., those between G_A_-G_A_ synthons) than those in the same cis-direction in form D (i.e., those between F_D_-F_D_ synthons). Of the five smallest Au:Au distances, the CN:CN distances in neighboring G_A_ synthons are within ±0.3 Å, and those of neighboring F_D_ synthons are within −0.1 Å to +1.0 Å. Furthermore, the surface area occupied by synthon G_A_ (approximately 160 Å^3^) is smaller than that occupied by synthon F_D_ (approximately 170 Å^3^), which, coupled with the energetically preferential formation of DFT-G_A_, could all lead to a dominant adsorption of form A on the Au surface.

**Table 5:** Comparing Distances between Neighboring Au Atoms in the Au(111) Surface and the CN–CN Distances Where the CN Bonds Lie in a Sterically Unimpeded Plane in the Bulk Crystals

		CN:CN distances (Å)		differences between Au:Au and CN:CN (Å)	
*N*th nearest Au-neighbor in Au-surface	Au:Au distances (Å)	form A	form D	form A	form D
1st	2.88	–	–	–	–
2nd	5.00	–	–	–	–
3rd	7.63	7.39	6.98	0.24	0.65
4th	10.40	10.12	11.41	–0.28	1.01
5th	12.57	12.43	12.51	0.14	–0.06

It should be noted that the DFT exploration of Au–entacapone interactions were simulated at 0 K and that the simulations were limited to snapshots of possible configurations due to their large computational expense. Hence, the next step was to explore if the predicted dominance of the adsorbed form A molecules at the terminated Au surface would persisted under more representative thermodynamic conditions.

### 4.6 Adsorption of Entacapone at the Au(111) Surface

There could be several different (and possibly simultaneously occurring) mechanisms for the adsorption and intermolecular assembly of entacapone molecules growing on the Au surface at the molecular level. For example, entacapone could adsorb at the Au surface layer-by-layer or by the assembly of clusters of interacting entacapone molecules on the surface, or by noninteracting molecules of entacapone encountering both the surface and one another concurrently.

#### 4.6.1 Layer-by-Layer Adsorption Model

In this interpretation, the individual entacapone molecules in solution react with, and adsorb to, the surface Au atoms creating an initial layer. Then, further individual entacapone molecules will interact with this first layer to form the second layer of entacapone molecules, and so on. This process was simulated using MD up to three layers with the initial configurations of form A molecules only (mindful that both form A and D molecules would be free to transform into one another during the simulation process).

Detailed analysis of the MD simulations with one layer of entacapone molecules (monolayer adsorption model) indicates that the simulated structures are much more similar to the form A molecular structure as evidenced by the lack of any molecules predicted from MD simulations in the RMSD range of 0.24–0.59 Å when overlapping with form D molecules (see further information in Supporting Information, Section S7.4 (Figure S15)). A similar trend was also found for the MD results with two and three layers of entacapone molecules. This further supports the findings from the DFT calculations, i.e., form A molecules being preferentially adsorbed on the Au surface.

It was also found that the orientation of the molecules within the single-layer simulations were almost always orientated parallel with respect to the aromatic ring plane to the Au surface, with higher similarity to the form A molecular structure than that of form D. The single-layer simulations also did not result in the formation of any of the synthonic dimers similar to the top synthons present in either of the form A or form D crystallographic structure, with the molecules within the layer having nearly identical normal distances to the Au surface.

In the subsequent two- and three- layer simulations, entacapone molecule–molecule interactions were found to overtake the molecule−Au interactions, where the molecular ordering within the adsorbed layer structure starts to show indications of forming synthon-like dimers. For example, dimers identified from two-layer MD simulations were found to be consistent with synthon E_A_ following simple rotational movement. Further details can be found in the Supporting Information, Section S7.4 (Figure S17).

Examination of the three-layer MD simulation produced a dimer similar to synthon A_A_ with only ∼3 Å difference in one translation, and also the synthon G_A_ could be reproduced after two small rotations (5° and 15°) of an identified dimer. However, the closest dimer structure to a synthon of form D requires more substantial motions to align the intermolecular interaction involving angular rotations of at least 15° and 40°. Therefore, the simulation data supports the higher probability of generating entacapone intermolecular dimers similar to the synthons from form A rather than those form D in an overall trend, that becomes more pronounced with an increasing number of molecular layers. Further details are given in the Supporting Information, Section S7.4 (Figure S18).

#### 4.6.2 Interacted and Uninteracted Trilayer Model

**Figure 12:**
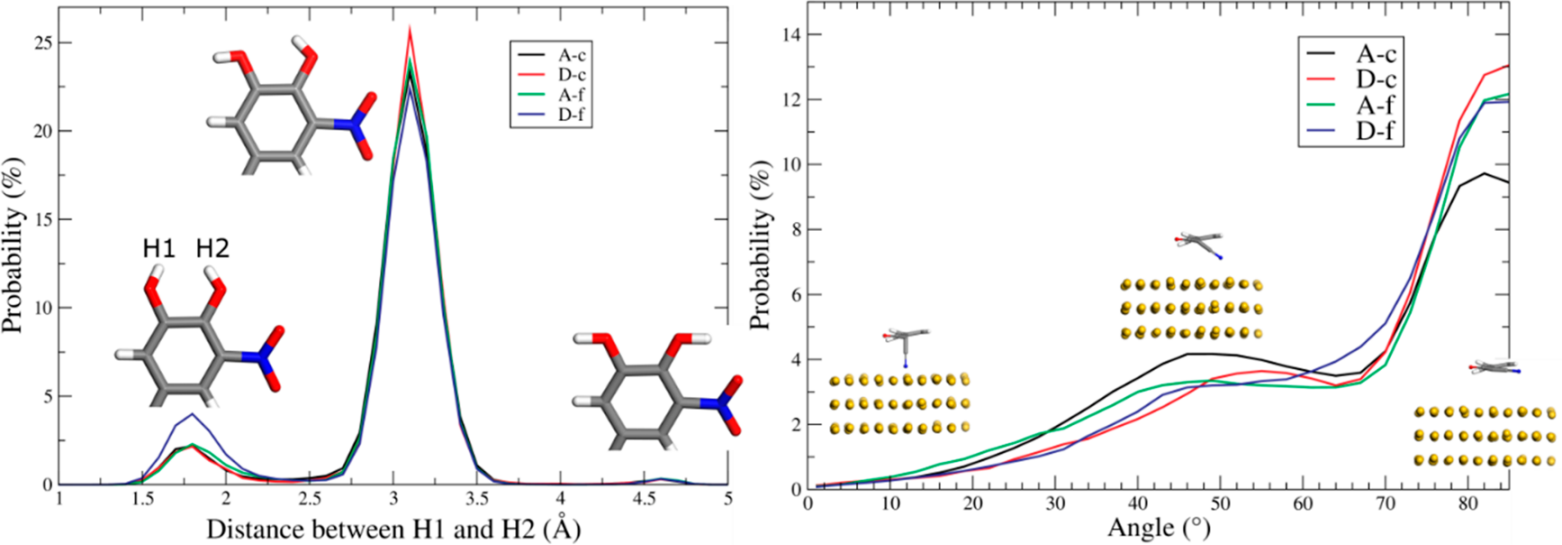
Probability distribution of (a) the distances between the OH–OH groups, with corresponding illustrations of their configurations, and (b) the angle between the cyano groups and the *z*-axis, with corresponding examples of their orientations. Note that the C≡N–Au angle is defined as 0° (C≡N perpendicular to Au surface) and 90° (C≡N parallel to Au surface). ‘A-c’ and ‘D-c’ mean entacapone molecules were placed close to the surface followed by a whole-system relaxation; ‘A-f’ and D-f’ mean entacopne molecules were allowed to interact, then translated to the Au surface, then the whole system was allowed to interact.

In a further scenario, entacapone was modeled in an interacted trilayer structure which was built based upon both form A and form D conformers, in which all three layers of entacapone molecules were free to interact, being initially constrained away from the presence of the Au(111) surface. Following this, the form A–A, and form D–D intermolecular interactions of the molecules were then free to interact with the Au surface. In another scenario, uninteracted trilayer models were built for both form A and form D, and all three layers of entacapone molecules were free to interact with one another and the surface from the start of the simulation. The results for forms A and D from these two methods are labeled in Figure 12 as (“A-c” and “D-c”) and (“A-f” and “D-f”), respectively. Full simulation details and simulation snapshots can be found in the Supporting Information, Section S7.4.

From an analysis of the MD trajectories, the distance between the two hydroxide hydrogen atoms representing the key difference between forms A and D was determined, (“H1” and “H2” in Figure 12a) revealing a peak between the hydroxide hydrogens at 3.1 Å, a distance that closely matches the form A-conformer and is consistent with the experimental observation that form A crystallizes at the Au surface. There are two smaller peaks at 4.6 and 1.8 Å. The former corresponds to the form D-conformer with two hydroxide hydrogens pointing in opposite directions. The latter does not represent conformations related to either forms A or D.

Also from the MD trajectories, an analysis of the orientation of the cyano group with respect to the surface, (Figure 12b) shows that the same trend was shared by the interacted and uninteracted trilayer models for both forms A and D: the majority of the entacapone molecules adsorb to the Au surface with their cyano group oriented parallel to the surface; some are angled between 30° to 50°, and other orientations have a negligible probability of occurring. Full simulation details with results and discussion can be found in the Supporting Information, Section S7.

## 5 Conclusions

The molecular, crystal, and surface chemistry of entacapone together with that for a template Au(111) has been investigated using molecular, crystallographic and surface modeling, to provide an insight into how the presence of this surface template promotes the formation of form A on the template surface, while concomitantly forming form D within the bulk solution.

The crystal structure of entacapone form D was solved and found to crystallize in an orthorhombic system (*Pna*2_1_) with eight molecules in the unit cell, and two molecules (different conformers) in the asymmetric unit. This is in contrast to the much simpler structure of form A. The molecules of form A have two intermolecular hydrogen bonds compared to one intermolecular and one intramolecular hydrogen bond for those of form D. The alkyl tail parts (amide and alkane groups) of the two form D molecules were positioned at ∼180° with respect to each other, while the tail part of a form A molecule roughly locates in between the two form D conformers, leading to a more planar-like molecular structure. The C≡N group presented a similar exposure for the molecules of both forms A and D.

The crystal chemistry of both forms A and D were found to be dominated by van der Waals and π–π stacking interactions with lower contributions (ca. 20%) from hydrogen bonding and electrostatic interactions, and having comparative lattice energies and convergence behavior consistent with their concomitant polymorphic behavior. The entacapone molecules in the form A structure were found to align themselves parallel to the {0–11} lattice planes, while in form D pairs of molecules align themselves alternately along the {0–11} and {011} planes in a more complex intermolecular packing structure with an interlocking criss-cross pattern.

Form D crystals were found to have a long, needle-like shape while form A has a more equant morphology, with the surface chemistry of the latter exposing the molecules’ cyano groups on its {010} and {011} habit faces. Simplicity, inherent by virtue of its low crystallographic symmetry, in the bulk crystal chemistry of form A permits the two molecules in the form A synthons to replicate each other by a simple translation/glide (Figure 8a), whereas the assembly of the form D synthons requires the combination of both rotation and translation/glide (Figure 8b). Reflecting the fact that the self-assembly of adsorbed entacapone molecules would be consistent with its surface nucleation, the data was found to be consistent with the parallel intermolecular orientation, as in the structure of form A, dominating surface adsorption and resulting in the preferential nucleation and growth of form A.

DFT calculations of entacapone binding energies to the Au substrate revealed that interactions between cyano functional groups of entacapone to Au dominate. The molecule/substrate distances with nearest neighbor Au atoms, were found to more closely match the distances between CN bonds oriented in the cis-direction in form A than those in the same cis-direction in form D. The larger binding energy of the form A dimer to the Au surface compared to that of the form D dimer, was found to be indicative of the preferential nucleation and growth based upon the formation of the form A dimer, supporting in turn a higher probability of entacapone adsorbing in its form A structure on the Au surface.

MD simulations confirmed that the majority of the entacapone molecules adsorb on the Au surface with their cyano group oriented parallel to the surface, with some being angled between 30° to 50°, while other orientations have a negligible probability of occurring. This supported the experimental observations, where crystals of entacapone form A were found to attach via their {010} surfaces to the Au(111) surface with their CN groups lying parallel to the Au surface. Trajectory analysis of the distance between the two hydroxide hydrogens which “fingerprint” the two polymorphs were found to more closely match the form A conformer, while the multilayer simulations indicated that the simulated structures were much more similar to the form A molecular structure, as evidenced by the lack of any molecules from MD simulations in the RMSD range of 0.24–0.59 Å when overlapping with form D molecules.

In conclusion, molecular modeling simulations using an integrated combination of empirical force fields, DFT and MD techniques revealed that the adsorption and orientation of entacapone form A synthons to the Au surface is both energetically and structurally more favorable than those of the form D synthons, thus providing, at the molecular-level, a robust explanation of the preferential nucleation of entacapone in form A on the Au template as experimentally characterized. The MD work to date though, was focused on solute binding and adsorption, and further studies are still needed to probe the role of solute concentration, solvent selection and the desolvation step in this template-driven polymorph direction process. The approach developed here has value in its extension to other systems where the molecular chemistry of some functional groups exhibit strong adhesion behavior in the presence of solution-treated Au surface templates and, through this, appropriate R&D workflows for characterizing templated crystallization systems can be envisaged.

## Supporting Information Available

Entacapone crystallization in the presence of a solution-treated Au(111) template (Section S1), crystal structure determination of entacapone form D (Section S2), molecular structure comparison and conformation analysis (Section S3), atomic coordinates and polarizability of entacapone molecules of forms A and D (Section S4), lattice energy, morphology, synthon analysis and crystal chemistry (Section S5), results and discussion—crystal morphology and surface chemistry (Section S6), DFT and MD simulations (Section S7), and parameters calculated (Section S8) (PDF)

## Dedication

^⊥^ Dedicated to the life and works of Professor John N. Sherwood.

## List of Symbols and Abbreviations

*a*, *b*, *c*: crystal unit cell parameters
*q*: atomic charge
*Z/Z′*: Number of molecules in the unit cell/asymmetric cell
α, β, γ: crystal unit cell parameters
AC: acceptor
B_A_, D_D_: synthon B of form A, synthon D of form D
CN: cyano
Coul: Coulombic energy contribution
CSD: Cambridge Structural Database
DFT: density functional theory
DN: donor
DN···AC: length between donor and acceptor
DN-H: bond length between donor and hydrogen
DN-H···AC: angle of donor–hydrogen–acceptor
H···AC: hydrogen bond length (acceptor and hydrogen)
MD: molecular dynamics
RMSD: root mean square deviation
vdW: van der Waals
