# Supplementary material for: Role of Molecular, Crystal, and Surface Chemistry in Directing the Crystallization of Entacapone Polymorphs on the Au(111) Template Surface ⊥
Source: Cryst Growth Des. 2023 May 1;23(6):4522–37. doi: 10.1021/acs.cgd.3c00294 (PMC10251417; doi:10.1021/acs.cgd.3c00294)
Supplement: Supplementary file 1 — cg3c00294_si_001.pdf [file cg3c00294_si_001.pdf]

Supporting Information

Role of Molecular, Crystal, and Surface Chemistry in Directing the Crystallisation of Entacapone Polymorphs on the Au(111) Template Surface §

Cai Y. Ma [1,], Dawn Geatches [2], Ya-Wen Hsiao [2], Ana Kwokal [3], Kevin J. Roberts [1]*

[1] *Centre for the Digital Design of Drug Products, School of Chemical and Process Engineering, University of Leeds, Leeds, LS2 9JT, UK*

[2] *Science and Technology Facilities Council, Daresbury Laboratory, Sci-Tech Daresbury, Warrington WA4 4AD, UK*

[3] *PLIVA Croatia Ltd., R&D, P. B. Filipovica 25, Zagreb 10000, Croatia; Present Address: Syngenta, Jealott's Hill International Research Centre, Bracknell RG42 6EY, U.K.*

§ Dedicated to the life and works of Professor John Sherwood

This supporting information provides supports to the main manuscript by providing further details of the followings: Experimental methods for entacapone crystallisation (Section S1); Crystal structure determination of entacapone form D (Section S2); Molecular structure comparison and conformation analysis (Section S3); Atomic coordinates and polarisability of entacapone molecules (Section S4); Lattice energy, morphology, synthon analysis and crystal chemistry (Section S5); Results and discussion: Crystal morphology and surface chemistry (Section 6); and DFT and MD simulations (Section S7).

S1. Entacapone Crystallisation in the Presence of a Solution-Treated Au(111) Template

The crystallisation experiments were carried out at room temperature using a clean Au(111) surface immersed close to a vertical position in the glass beaker with the supersaturated entacapone solution without stirring [1]. A binary solvent system was used: 88 % (vol/vol) of the distilled water and 12% (vol/vol) of the acetone (Acros Organic). The solutions were prepared by the dissolution of the Entacapone in acetone, followed by the addition of water of the desired concentrations (0.3 g/L). The vessel was covered, and left undisturbed, under ambient conditions, for about 12 hours. In order to determine orientation of the resultant crystals on the templates, they were removed from the template surface by peeling the thin layer of mica with the gold film still attached to the crystal.

S2. Crystal Structure Determination of Entacapone Form D

Table S1. Refinement details used for the Entacapone form D crystal structure determination.

Refinement	Least-squares matrix	$R[F^2 > 2\sigma(F^2)]$	$wR(F^2)$	S	$(\Delta/\sigma)_{max}$	$\Delta\rho_{max}$	$\Delta\rho_{min}$
F^2	Full	0.030	0.062	0.98	0.001	0.11 e Å^{-3}	−0.10 e Å^{-3}

Table S2. Crystal data and structure refinement for entacapone Form D.

**Items**	**Details**
Archive code	data_exp_409
Formula	$C_{14} H_{15} N_3 O_5$
Formula weight	305.29 g mol^{-1}
Size	0.5 × 0.2 × 0.2 mm
Crystal morphology Colourless	needle
Temperature	297(2) K
Wavelength	1.5418 Å [CuKa]
Crystal system	Orthorhombic
Space group	P n a 2_1
Unit cell dimensions	a = 15.1885(12) Å, b = 25.678(3) Å, c = 7.4958(8) Å
	α = 90°, β = 90°, γ = 90°
Unit cell volume	2930.5(5) Å^3
Z (Z')	4 (2)
Density (calculated)	1.392 mg m^{-3}
Absorption coefficient	0.99 mm^{-1}
F(000)	1288
Data collection range	3.38° ≤ 2θ ≤ 61.4°
Index ranges	$-15 \le h \le 17$, $-28 \le k \le 26$, $-8 \le l \le 7$
Reflections collected	9317
Independent reflections	2394 [R_{int} = 0.0688]
Observed reflections	1127 [$I > 2\sigma(I)$]
Absorption correction	Multi-scan
Max. and min. transmission	0.834 and 0.805
Refinement method	Full
Data/restraints/parameters	2394/1/402
Goodness of fit	0.978
Final R indices [$I > 2\sigma(I)$]	R_1 = 0.0303, wR_2 = 0.0487
R indices (all data)	R_1 = 0.0843, wR_2 = 0.0621

The crystal packing structure of entacapone Form D has a hydrogen bond between the first molecule (stick) and the second one (green coloured) as shown in **Figure S1**. This crystal presents two hydrogen-bonding networks and overlays each other.

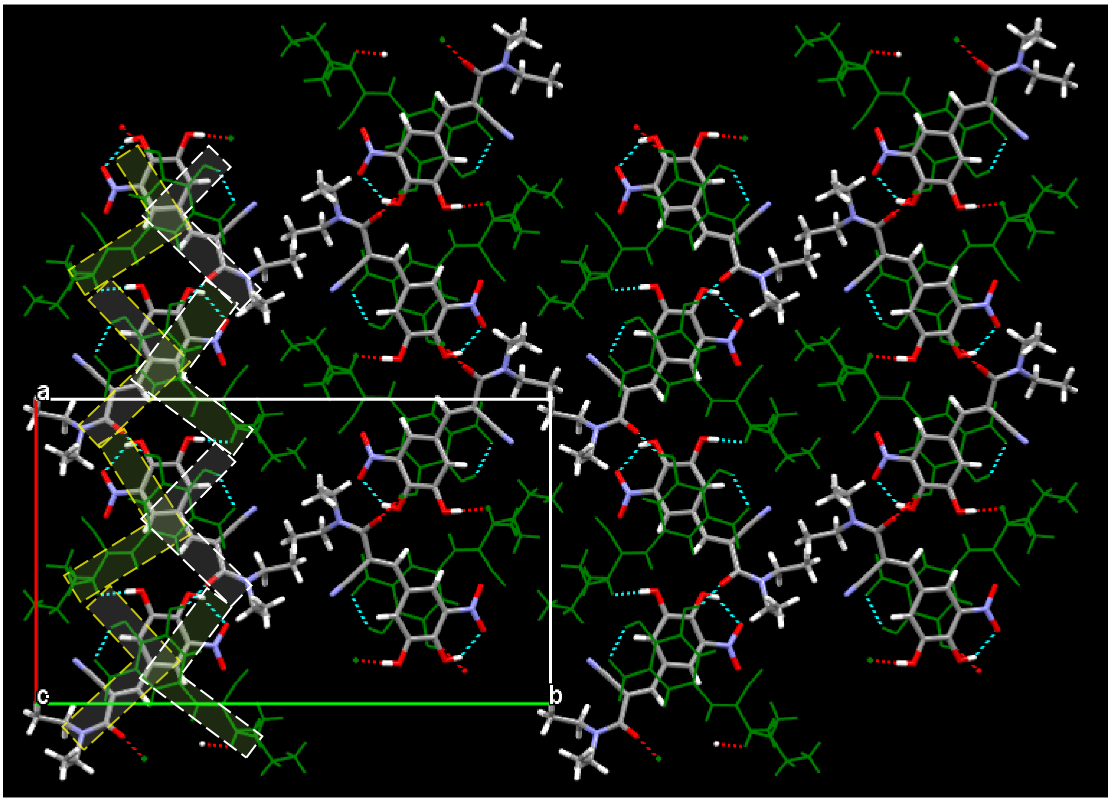

Figure S1. Crystal packing diagram of entacapone Form D crystal (view 2 × 2 × 2) showing that one molecule (stick) of Form D interacts via hydrogen bonding (blue dotted line) with the second molecule (green coloured) of Form D to form two twisted hydrogen networks (yellow and white dashed blocks).

S3. Molecular Structure Comparison and Conformation Analysis

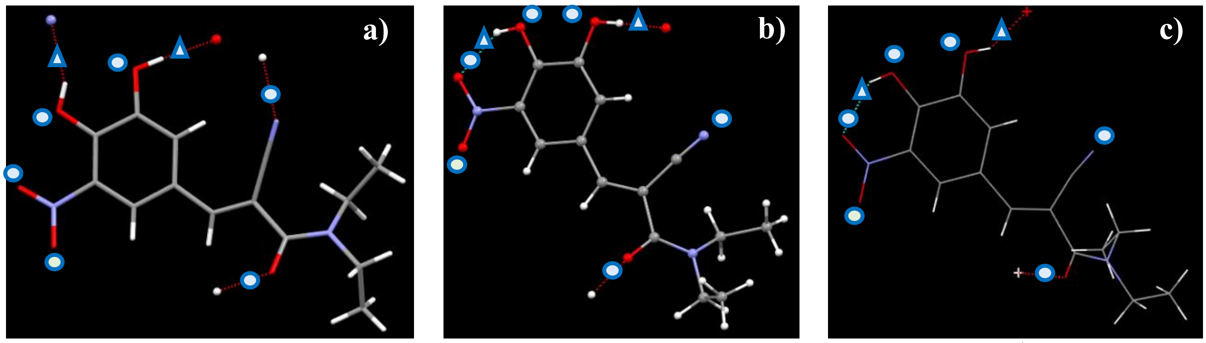

Figure S2. The molecular structures of entacapone a) polymorph form A; b) form D (1st molecule) and c) form D (2nd molecule) with their hydrogen bonding donors (triangle) and acceptors (circle).

By comparing their molecules in their asymmetric units, as shown in **Figure S3**, both the first and second molecules in the asymmetric units of form D in this study and form II [2] are overlaid well for the aromatic ring, phenol, nitro and alkene structures. However, the rest of the molecular structures (cyano, amide and alkane) between the first molecules from form D and form II are mirrored with about 10°, 60° and 57°, respectively, against a mirror plane (pink coloured, **Figure S3**(a)) of the overlaying aromatic ring, phenol, nitro and alkene. Similarly, the second molecules from form D and form II [2] overlay well for the aromatic ring and the surrounding structures and also the cyano, amide and alkane structures are mirrored against a plane (pink coloured in **Figure S3**(c)) with estimated

angles of 9°, 50° and 40°, respectively.

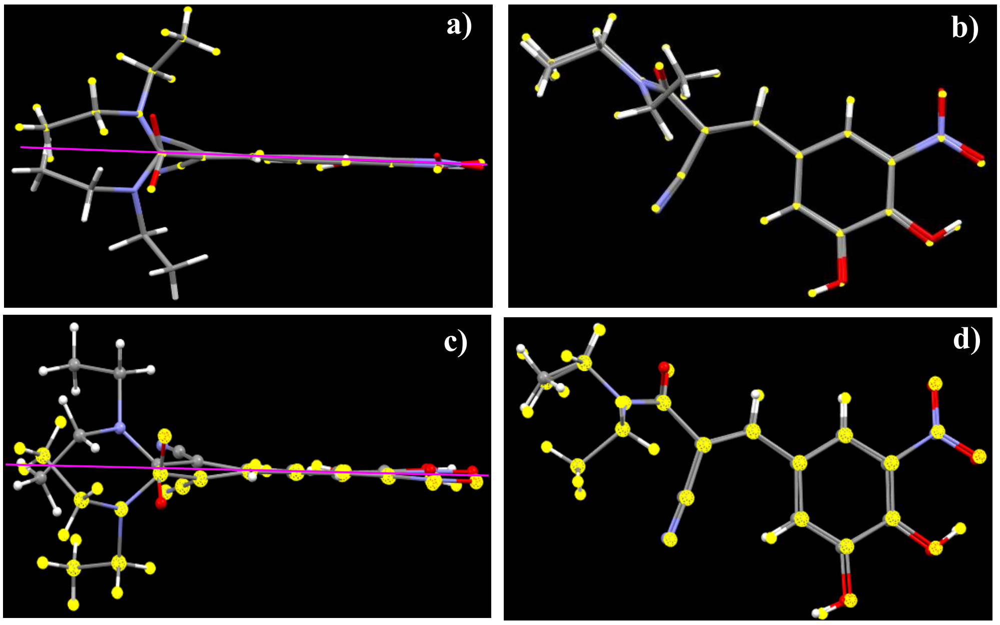

Figure S3. The molecular structure comparisons of entacapone polymorph form D and form II [2]: a&b) first molecules (stick); c&d) second molecules (ball and stick). The molecules of form II are plotted with yellow dots.

Figure S4 shows the molecular structures of form A and form D. As shown in **Figure S4**(e&f), the two molecules in the asymmetric unit of form D are not identical. Both molecules have very similar coordinates for their head parts (including phenol, nitro, aromatic ring and alkene) and alkene and cyano functional groups, but the tail part (cyano, amide and alkane groups) of the second molecule (wire) is about 180° rotation of the first one (stick and ball). Comparing the form A molecule with the first molecule (**Figure S4**(a&b)) and the second molecule (**Figure S4**(c&d)) of form D, the head part has similar coordinate except the hydrogen in one of the phenol groups in the form A molecule which is about 180° rotation. The first molecule of form D has similar structure to the form A molecule (**Figure S4**(a&b)), hence the tail part of the second molecule of form D also has about 180° rotation comparing form A molecule (**Figure S4**(c&d)).

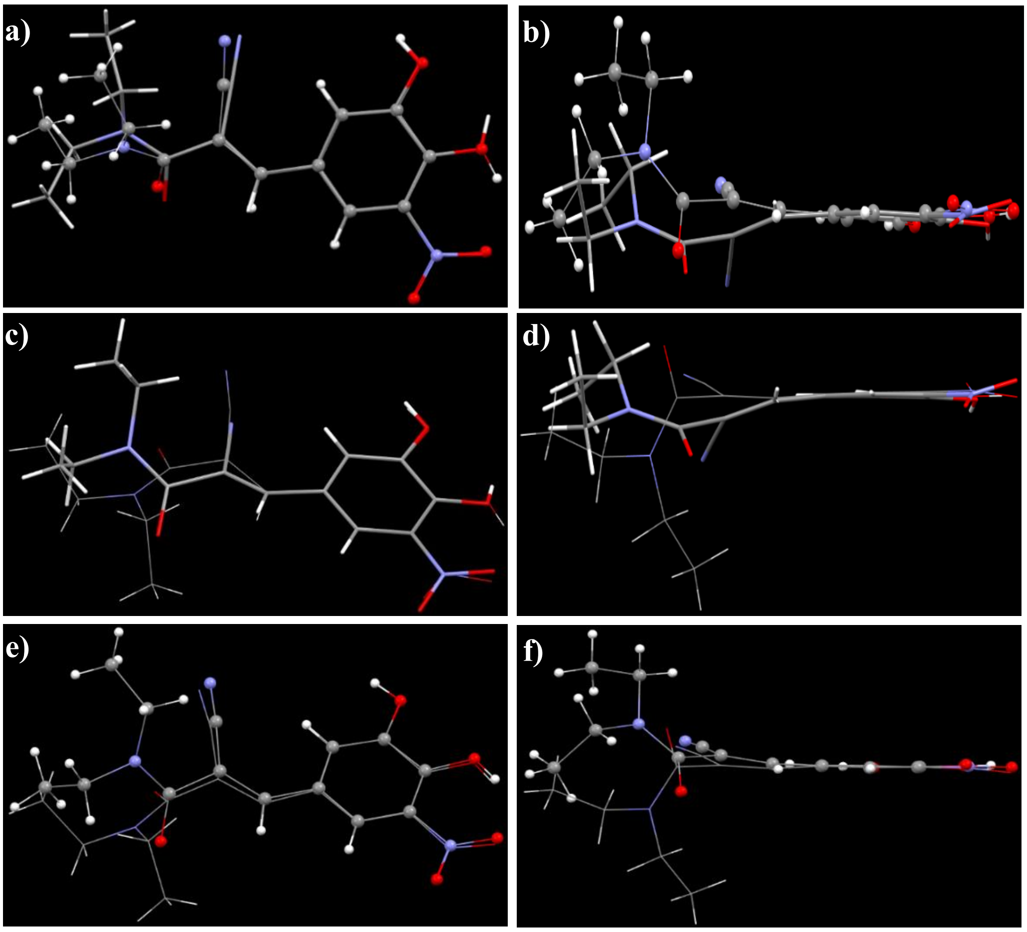

Figure S4. The comparisons of the molecular structures of entacapone: a) & b) polymorph form A (stick), form D, first molecule (stick and ball); c) & d) form A (stick), form D, second molecule (wire); e) & f) form D, first (stick and ball) and second (wire) molecules.

Figure S5 shows the definitions of the identified torsions and their corresponding torsion angles in the molecule of form A and the two molecules of form D. From the comparisons of the three molecular structures as shown in **Figure S4**, the existence of the tail part rotations and the importance of cyano functional group for the possible interactions with Au surface led to the selection of torsion number 5 (C2-C1-C7-C8 in form A molecule, C15-C14-C17-C18 in the first molecule of form D, C25-C24-C27-C28 in the second molecule of form D).

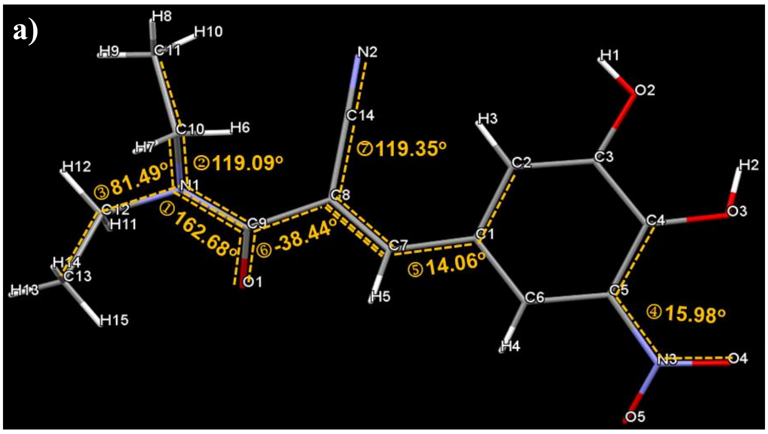

Torsion	Angle (°)
C10-N1-C9-O1 (1)	162.68
C9-N1-C10-C11 (2)	119.09
C9-N1-C12-C13 (3)	81.49
O4-N3-C5-C4 (4)	15.98
C2-C1-C7-C8 (5)	14.06
C7-C8-C9-O1 (6)	-38.44
C7-C8-C14-N2 (7)	119.35

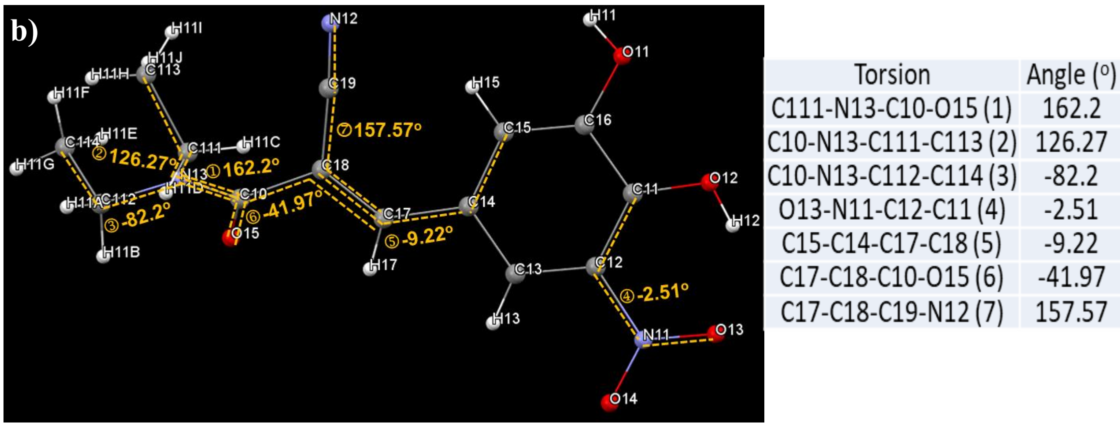

Torsion	Angle (°)
C111-N13-C10-O15 (1)	162.2
C10-N13-C111-C113 (2)	126.27
C10-N13-C112-C114 (3)	-82.2
O13-N11-C12-C11 (4)	-2.51
C15-C14-C17-C18 (5)	-9.22
C17-C18-C10-O15 (6)	-41.97
C17-C18-C19-N12 (7)	157.57

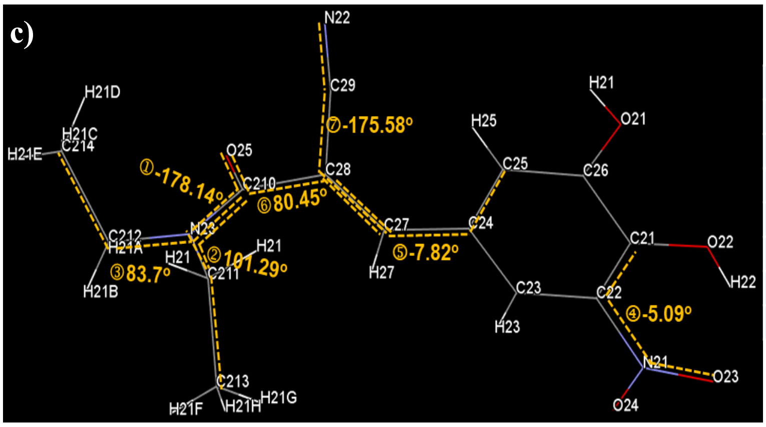

Torsion	Angle (°)
C211-N23-C210-O25 (1)	-178.14
C210-N23-C211-C213 (2)	101.29
C210-N23-C212-C214 (3)	83.7
O23-N21-C22-C21 (4)	-5.09
C25-C24-C27-C28 (5)	-7.82
C27-C28-C210-O25 (6)	80.45
C27-C28-C29-N22 (7)	-175.58

Figure S5. The definitions of the entacapone molecular torsions: a) Form A; b) Form D, first molecule; c) Form D, second molecule.

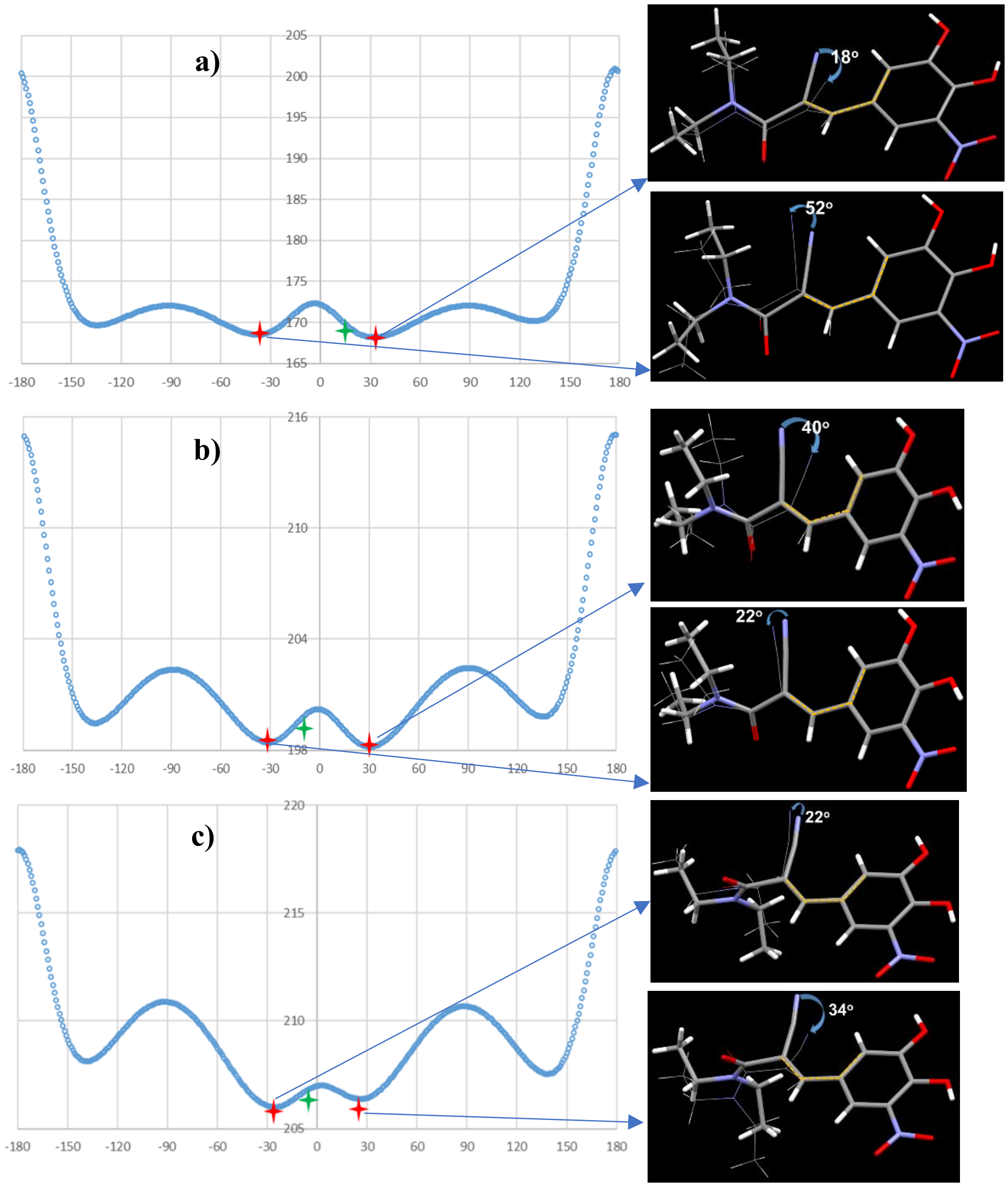

Figure S6. The energy variations with the torsion angles and the energetically-ranked top two molecular structures of entacapone crystals: a) Form A (Torsion 5: C2-C1-C7-C8, Red symbols: top 2 torsion angles (32.06° & -37.94°) as stick, Green symbol: angle (14.06°) in crystal structure as wire); b) Form D, first molecule (Torsion 5: C15-C14-C17-C18, Red symbols: top 2 torsion angles (30.78° & -31.22°) as stick, Green symbol: angle (-9.22°) in crystal structure as wire); c) Form D, second molecule (Torsion 5: C25-C24-C27-C28, Red symbols: top 2 torsion angles (-25.82° & 26.18°) as stick, Green symbol: angle (-7.82°) in crystal structure as wire).

The energy variations with the torsion angles (-180° to +180°) of form A molecule and two form D molecules and the energetically-ranked top two molecular structures are plotted in **Figure S6**. The corresponding torsion angles for the molecular structures extracted directly from the crystal structures of forms A and D of entacapone are shown as green symbols. These molecular structures (plotted as wire) were compared with those two top-ranked structures (plotted as stick). It was found that the two top-ranked molecular structures are symmetrically located at the left and right sides of an energetic maximum with a torsion angle of -2.94°, -0.22° and 0.18° for the form A molecule and the first and

second molecules of form D, respectively. The top-ranked two torsion angles for the form A molecule are 32.06° and -37.94° with the corresponding differences against the torsion angle (14.06°) of the molecule from form A crystal structure being 18° and 52° (**Figure S6**(a)). Similarly the torsion angle differences between the top-ranked molecules and the molecules from crystal structure of form D are 40° and 22° for the first molecule (**Figure S6**(b), 22° and 34° for the second molecule (**Figure S6**(c)).

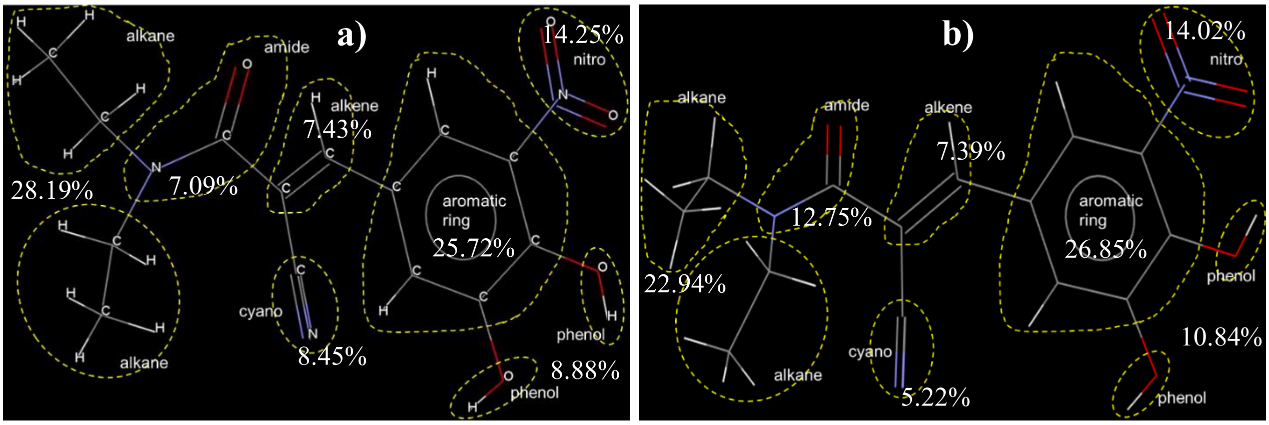

Figure S7. The definitions of functional groups – alkane, alkene, amide, aromatic ring, cyano, nitro and phenol: a) form A; b) form D.

S4. Atmoic Coordinates and Polarisability of Entacapone Molecules of Forms A and D

Table S3. Atomic coordinates of molecules from Entacapone Forms A and D.

Form A atoms	Form D atoms		Form A			Form D (1st molecule)			Form D (2nd molecule)		
	1st molecule	2nd molecule	x	y	z	x	y	z	x	y	z
**O1**	O1 (O25)	O10 (O15)	1.4062	0.7399	1.0699	0.8573	0.3786	0.7622	0.3929	0.333	0.7089
**O2**	O3 (O21)	O6 (O11)	0.3845	0.5322	0.8571	1.3104	0.2606	0.5394	0.8698	0.2939	0.9631
**H1**	H2 (H21)	H16 (H11)	0.3916	0.594	0.9295	1.3188	0.2897	0.5809	0.8672	0.323	0.9181
**O3**	O2 (O22)	O8 (O12)	0.3947	0.3616	0.6221	1.2693	0.1727	0.4009	0.8587	0.2047	1.122
**H2**	H1 (H22)	H19 (H12)	0.3203	0.36	0.6761	1.2504	0.1456	0.3579	0.8487	0.1766	1.1701
**O4**	O4 (O23)	O7 (O13)	0.5807	0.3038	0.4261	1.1444	0.1135	0.2837	0.7574	0.1302	1.2242
**O5**	O5 (O24)	O9 (O14)	0.8708	0.3394	0.4812	1.0079	0.1339	0.3245	0.6159	0.1358	1.1983
**N1**	N1 (N23)	N5 (N13)	1.3318	0.9267	1.2066	0.8915	0.4286	0.5258	0.4021	0.4103	0.8478
**N2**	N3 (N22)	N6 (N12)	0.8852	0.7451	1.2365	1.0867	0.1444	0.3347	0.6404	0.4238	0.8029
**N3**	N2 (N21)	N4 (N11)	0.7207	0.3555	0.508	1.1128	0.4122	0.7838	0.6896	0.1532	1.175
**C1**	C8 (C24)	C16 (C14)	0.8669	0.6145	0.8419	1.0688	0.2777	0.5181	0.6293	0.2773	0.9525
**C2**	C6 (C25)	C18 (C15)	0.7026	0.6181	0.8916	1.1585	0.2894	0.5523	0.7123	0.3002	0.9302
**H3**	H5 (H25)	H18 (H15)	0.6996	0.6781	0.9779	1.1736	0.3215	0.6006	0.7164	0.3333	0.8807
**C3**	C7 (C26)	C23 (C16)	0.548	0.5362	0.8165	1.2242	0.2533	0.5146	0.7882	0.2745	0.9805
**C4**	C2 (C21)	C19 (C11)	0.5471	0.4442	0.6893	1.201	0.2049	0.4389	0.7829	0.2258	1.0669
**C5**	C10 (C22)	C17 (C12)	0.7099	0.4449	0.64	1.1135	0.1940	0.4106	0.6986	0.2039	1.0867
**C6**	C4 (C23)	C15 (C13)	0.8675	0.5289	0.7142	1.0475	0.2298	0.447	0.6228	0.2284	1.029
**H4**	H3 (H23)	H17 (H13)	0.9755	0.5278	0.6774	0.989	0.2215	0.4236	0.5682	0.2123	1.0414
**C7**	C5 (C27)	C20 (C17)	1.0405	0.6898	0.9184	0.9958	0.3143	0.5457	0.5462	0.3023	0.9023
**H5**	H4 (H27)	H20 (H17)	1.1323	0.6958	0.8642	0.9416	0.3027	0.5036	0.4975	0.2804	0.9084
**C8**	C1 (C28)	C21 (C18)	1.0912	0.7523	1.0535	0.9941	0.3609	0.6204	0.5276	0.3509	0.8492

**C9**	C3 (C210)	C22 (C10)	1.2886	0.8085	1.1106	0.9071	0.3900	0.6388	0.4347	0.3645	0.7986
**C10**	C13 (C211)	C26 (C111)	1.2106	1.0341	1.234	0.9481	0.4426	0.3729	0.4362	0.4437	0.992
**H6**	H11 (H211C)	H23 (H11C)	1.095	1.0051	1.1666	1.0064	0.4283	0.3922	0.4898	0.4284	1.039
**H7**	H12 (H211D)	H24 (H11D)	1.266	1.1251	1.2174	0.9535	0.4802	0.3676	0.3934	0.4449	1.0878
**C11**	C14 (C213)	C28 (C113)	1.1713	1.0568	1.3806	0.9136	0.4232	0.1973	0.4558	0.4988	0.9318
**H8**	H13 (H21F)	H28 (H11H)	1.0903	1.1292	1.3912	0.8551	0.4364	0.1787	0.4043	0.5134	0.8767
**H9**	H14 (H21G)	H29 (H11I)	1.2845	1.0882	1.4482	0.9122	0.3858	0.1981	0.5033	0.4984	0.8475
**H10**	H15 (H21H)	H30 (H11J)	1.1134	0.9679	1.3974	0.9513	0.435	0.1029	0.472	0.5196	1.0331
**C12**	C9 (C212)	C25 (C112)	1.5251	0.9682	1.2715	0.8116	0.4597	0.5509	0.3132	0.4237	0.7831
**H11**	H6 (H21A)	H21 (H11A)	1.5791	0.8824	1.2879	0.7641	0.4372	0.5898	0.2871	0.4492	0.8625
**H12**	H7 (H21B)	H22 (H11B)	1.5342	1.0312	1.3634	0.7947	0.4752	0.4379	0.2766	0.3927	0.7858
**C13**	C11 (C214)	C27 (C114)	1.633	1.0434	1.1833	0.8255	0.5025	0.6879	0.3146	0.4451	0.5969
**H13**	H8 (H21C)	H25 (H11E)	1.7592	1.068	1.2316	0.8733	0.5244	0.6508	0.3468	0.4219	0.5205
**H14**	H9 (H21D)	H26 (H11F)	1.5824	1.1299	1.1685	0.8392	0.4872	0.8014	0.3426	0.4786	0.5972
**H15**	H10 (H21E)	H27 (H11G)	1.6272	0.9809	1.0929	0.7728	0.5229	0.6981	0.2554	0.4486	0.5537
**C14**	C12 (C29)	C24 (C19)	0.9741	0.7498	1.1537	1.0645	0.3869	0.7089	0.5923	0.3909	0.8266

Table S4. Polarisability of molecules from Entacapone Forms A and D (q_A – atomic charge of Form A molecule; q_{D1} – atomic charge of Form D molecule; q_{D2} – atomic charge of Form D molecule).

**Form A atoms**	**Form D atoms**		**q_A /ecu**	**q_{D1} /ecu**	**q_{D2} /ecu**	**(q_A-q_{D1}) /ecu**	**(q_A-q_{D2}) /ecu**	**(q_{D1}-q_{D2}) /ecu**
	**1st molecule**	**2nd molecule**						
**O1**	O1 (O25)	O10 (O15)	-0.3619	-0.39190	-0.35640	0.03	-0.0055	-0.0355
**O2**	O3 (O21)	O6 (O11)	-0.2504	-0.19200	-0.21730	-0.0584	-0.0331	0.0253
**H1**	H2 (H21)	H16 (H11)	0.2265	0.21380	0.23690	0.0127	-0.0104	-0.0231
**O3**	O2 (O22)	O8 (O12)	-0.1765	-0.19010	-0.18420	0.0136	0.0077	-0.0059
**H2**	H1 (H22)	H19 (H12)	0.2353	0.23720	0.23860	-0.0019	-0.0033	-0.0014
**O4**	O4 (O23)	O7 (O13)	-0.3230	-0.38610	-0.39730	0.0631	0.0743	0.0112
**O5**	O5 (O24)	O9 (O14)	-0.3609	-0.31950	-0.31920	-0.0414	-0.0417	-0.0003
**N1**	N1 (N23)	N5 (N13)	-0.3227	-0.31730	-0.33220	-0.0054	0.0095	0.0149
**N2**	N3 (N22)	N6 (N12)	-0.0381	-0.01530	-0.04290	-0.0228	0.0048	0.0276
**N3**	N2 (N21)	N4 (N11)	0.5545	0.53360	0.56370	0.0209	-0.0092	-0.0301
**C1**	C8 (C24)	C16 (C14)	-0.0910	-0.07200	-0.09020	-0.019	-0.0008	0.0182
**C2**	C6 (C25)	C18 (C15)	-0.0936	-0.11880	-0.10420	0.0252	0.0106	-0.0146
**H3**	H5 (H25)	H18 (H15)	0.1365	0.13450	0.14550	0.002	-0.009	-0.011
**C3**	C7 (C26)	C23 (C16)	-0.0199	0.06140	0.06540	-0.0813	-0.0853	-0.004
**C4**	C2 (C21)	C19 (C11)	0.1710	0.16060	0.15570	0.0104	0.0153	0.0049
**C5**	C10 (C22)	C17 (C12)	-0.1487	-0.18660	-0.20170	0.0379	0.053	0.0151
**C6**	C4 (C23)	C15 (C13)	-0.0124	-0.02730	-0.02260	0.0149	0.0102	-0.0047
**H4**	H3 (H23)	H17 (H13)	0.1575	0.14830	0.14840	0.0092	0.0091	-0.0001
**C7**	C5 (C27)	C20 (C17)	0.0146	0.00400	0.02180	0.0106	-0.0072	-0.0178
**H5**	H4 (H27)	H20 (H17)	0.1379	0.12370	0.12920	0.0142	0.0087	-0.0055
**C8**	C1 (C28)	C21 (C18)	-0.0846	-0.08770	-0.10030	0.0031	0.0157	0.0126
**C9**	C3 (C210)	C22 (C10)	0.3478	0.36450	0.35620	-0.0167	-0.0084	0.0083

**C10**	C13 (C211)	C26 (C111)	0.0017	0.00800	0.00920	-0.0063	-0.0075	-0.0012
**H6**	H11 (H211C)	H23 (H11C)	0.0825	0.08310	0.08430	-0.0006	-0.0018	-0.0012
**H7**	H12 (H211D)	H24 (H11D)	0.0795	0.08760	0.07490	-0.0081	0.0046	0.0127
**C11**	C14 (C213)	C28 (C113)	-0.2060	-0.20450	-0.19950	-0.0015	-0.0065	-0.005
**H8**	H13 (H21F)	H28 (H11H)	0.0811	0.07680	0.07260	0.0043	0.0085	0.0042
**H9**	H14 (H21G)	H29 (H11I)	0.0748	0.07230	0.07970	0.0025	-0.0049	-0.0074
**H10**	H15 (H21H)	H30 (H11J)	0.0837	0.08230	0.07860	0.0014	0.0051	0.0037
**C12**	C9 (C212)	C25 (C112)	-0.0032	-0.00350	-0.00220	0.0003	-0.001	-0.0013
**H11**	H6 (H21A)	H21 (H11A)	0.1007	0.10590	0.07640	-0.0052	0.0243	0.0295
**H12**	H7 (H21B)	H22 (H11B)	0.0800	0.07890	0.09120	0.0011	-0.0112	-0.0123
**C13**	C11 (C214)	C27 (C114)	-0.2106	-0.19550	-0.19750	-0.0151	-0.0131	0.002
**H13**	H8 (H21C)	H25 (H11E)	0.0799	0.06330	0.08330	0.0166	-0.0034	-0.02
**H14**	H9 (H21D)	H26 (H11F)	0.0653	0.08320	0.06560	-0.0179	-0.0003	0.0176
**H15**	H10 (H21E)	H27 (H11G)	0.0874	0.08130	0.07600	0.0061	0.0114	0.0053
**C14**	C12 (C29)	C24 (C19)	-0.0951	-0.09620	-0.08550	0.0011	-0.0096	-0.0107

S5. Lattice Energy, Morphology, Synthon Analysis and Crystal Chemistry

S5.1 Intermolecular interaction and lattice energy

The intermolecular pair interaction energies together for the two polymorphic structures were calculated using HABIT98 (an enhanced version of HABIT95 [3], which was developed from HABIT [4]) with Dreiding forcefield [5] and MOPAC [6] atomic charges. The energies were as partitioned into their constituent components (van der Waals, hydrogen bond and electrostatic) and their 3D spatial arrangement within the crystal lattice was characterised using Materials Studio. The crystal lattice energy due to inter-molecular packing interactions was summed as a function of radial distance. In this, the convergence was tested by increasing the intermolecular summation sphere radius of calculation to 30 Å using a step size of 1 Å with the data displayed using both cumulative and discretised radial interaction energy plots. The relative contributions of the individual atoms within the molecules were assessed through partitioning the lattice energy onto of the different function groups. Through this, the structural chemistry and intermolecular energy of all the constituent inter-molecular interactions (synthons) were characterised, classified and ranked [7-10].

S5.2 Morphology prediction and synthon analysis

The morphologically-important faces associated with their growth layer thickness (d_{hkl}) were identified and ranked by the BFDH method [11-14] using Mercury [10]. Dominant intermolecular interactions identified in the lattice energy calculations were partitioned between the intrinsic synthons which were fully coordinated within the growth layer (surface stability) (E_{sl}^{hkl}) and the extrinsic (growth promoting) synthons (E_{att}^{hkl}) associated with surface termination by the external morphology as summarised in Eq. (1):

$$E_{cryst} = E_{att}^{hkl} + E_{sl}^{hkl} \quad (1)$$

The relative growth rate of each crystal habit face was taken as being proportional to E_{att}^{hkl} [15] which

was normalised with respect to the lowest growth rate and a Wulff plot [16] was used to project the predicted crystal morphology for each of the polymorphic forms. Additionally, the surface anisotropy factor (ζ_{hkl}), identifying the degree of synthon saturation for the crystal surfaces (*hkl*), was calculated using Eq. (2):

$$\zeta_{hkl} = E_{sl}^{hkl} / E_{cryst}^{hkl} \qquad (2)$$

S5.3 Assessment of surface chemistry

The intermolecular chemistry of the selected crystal growth slices and their surface chemistry, together with their constituent synthons, were visualised using Materials Studio and tabulated on a face-specific basis.

The surface energy of the selected crystal surfaces (*hkl*) were calculated from the surface attachment energy using Eq. (3):

$$\gamma_{hkl} = \frac{Z\ E_{att}\ d_{hkl}}{2\ V_{call}\ N_A} \qquad (3)$$

where Z is the number of molecules in the unit cell, N_A is Avogadro's number, V_{cell} is the crystallographic unit cell volume.

The overall particle surface energy ($\gamma_{particle}$) for each polymorph was estimated by calculating surface-area (SA_{hkl}) weighted average of the calculated surface energies based on the predicted morphology, using Eq. (4):

$$\gamma_{particle} = \sum \frac{Z\ E_{att}\ d_{hkl}}{2\ V_{cell}\ N_A}\ M_{hkl}\ SA_{hkl} \qquad (4)$$

where M_{hkl} is the multiplicity of crystal form. The fractional surface area of the habit faces (*hkl*) was calculated using Mercury [10].

The inter-planar surface roughness/smoothness or rugosity of the selected crystal surfaces representing the atomic variation in height with respect to a given crystallographic plane, were calculated by taking the root mean squared variation of all the atomic positions within the asymmetric unit with respect to the surface of the crystal plane (*hkl*).

Table S5 summarises the relative contributions of van der Waals, Coulombic and hydrogen-bond energies to the total lattice energy of entacapone forms A and D with DFT calculations showing clearly larger value for form A. It can be seen that form A has a slightly larger lattice energy than form D. Both forms are dominated by van der Waals interactions with the contributions of 79.19% and 79.28% to the total lattice energies of form A and form D, respectively. However, form A has a higher hydrogen-bond contribution to its lattice energy than form D (13.28% and 4.66%, respectively) and form D possess a greater electrostatic contribution than form A (16.06% and 7.53%, respectively).

Table S5. Details of the relative contribution of vdW, Coulombic energy and H-bond energy to the total lattice energy of entacapone forms A and D.

Type	Form A	Form D	Percentage contribution to lattice energy %	
			Form A	Form D
**vdW (kcal/mol)**	-28.92	-28.73	79.19	79.28
**Coulombic forces (kcal/mol)**	-2.75	-5.82	7.53	16.06
**H-bond (kcal/mol)**	-4.85	-1.69	13.28	4.66
**Lattice Energy (kcal/mol)**	-36.52	-36.24	100	100
**DFT Lattice Energy (kcal/mol)**	-52.24	-44.87		

The definitions of molecular fragments (functional groups) for the molecules of forms A and D in their asymmetric units are displayed in **Figure S7**. There are seven functional groups existing in the entacapone molecule: phenol (×2), nitro, aromatic ring, alkene, cyano, amide, alkane (×2). The nitro, amide, cyano and two phenol groups have hydrogen-bond acceptors and/or donors, and may be involved in hydrogen-bond interactions. **Table S6** lists the functional group contributions of entacapone form A and form D to their total lattice energies. For both forms A and D, the aliphatic (including one alkene and two alkane) and aromatic ring groups make contributions of 61.34% (35.62% for aliphatic and 25.72% for aromatic rings) and 57.18% (30.33% for aliphatic and 26.85% for aromatic rings) to the corresponding lattice energies, respectively, demonstrating the dominance of dispersive interactions in the crystal lattice.

Table S6. The relative contributions of the molecular fragments to the lattice energy of entacapone forms A and D.

Functional Group	Dispersive energy (kcal/mol)		Electrostatic energy (kcal/mol)		Total energy (kcal/mol)		% contribution to lattice energy	
	Form A	Form D	Form A	Form D	Form A	Form D	Form A	Form D
**Aliphatic (2Alkane+Alkene)**	-11.62	-10.35	-1.39	-0.64	-13.01	-10.99	35.62	30.33
**Aromatic ring**	-8.38	-9.42	-1.01	-0.31	-9.39	-9.73	25.72	26.85
**nitro**	-3.43	-3.77	-0.38	-1.31	-3.81	-5.08	14.25	14.02
**Phenol (×2)**	-1.44	-2.13	-1.80	-1.80	-3.24	-3.93	8.88	10.84
**Cyanide**	-3.08	-1.74	-0.01	-0.15	-3.09	-1.89	8.45	5.22
**Amide**	-1.29	-1.70	-1.29	-2.92	-2.58	-4.62	7.09	12.75
**Total**	-29.00	-29.11	-7.52	-7.13	-36.52	-36.24	100	100
**%**	79.41	80.33	20.59	19.67	100	100		

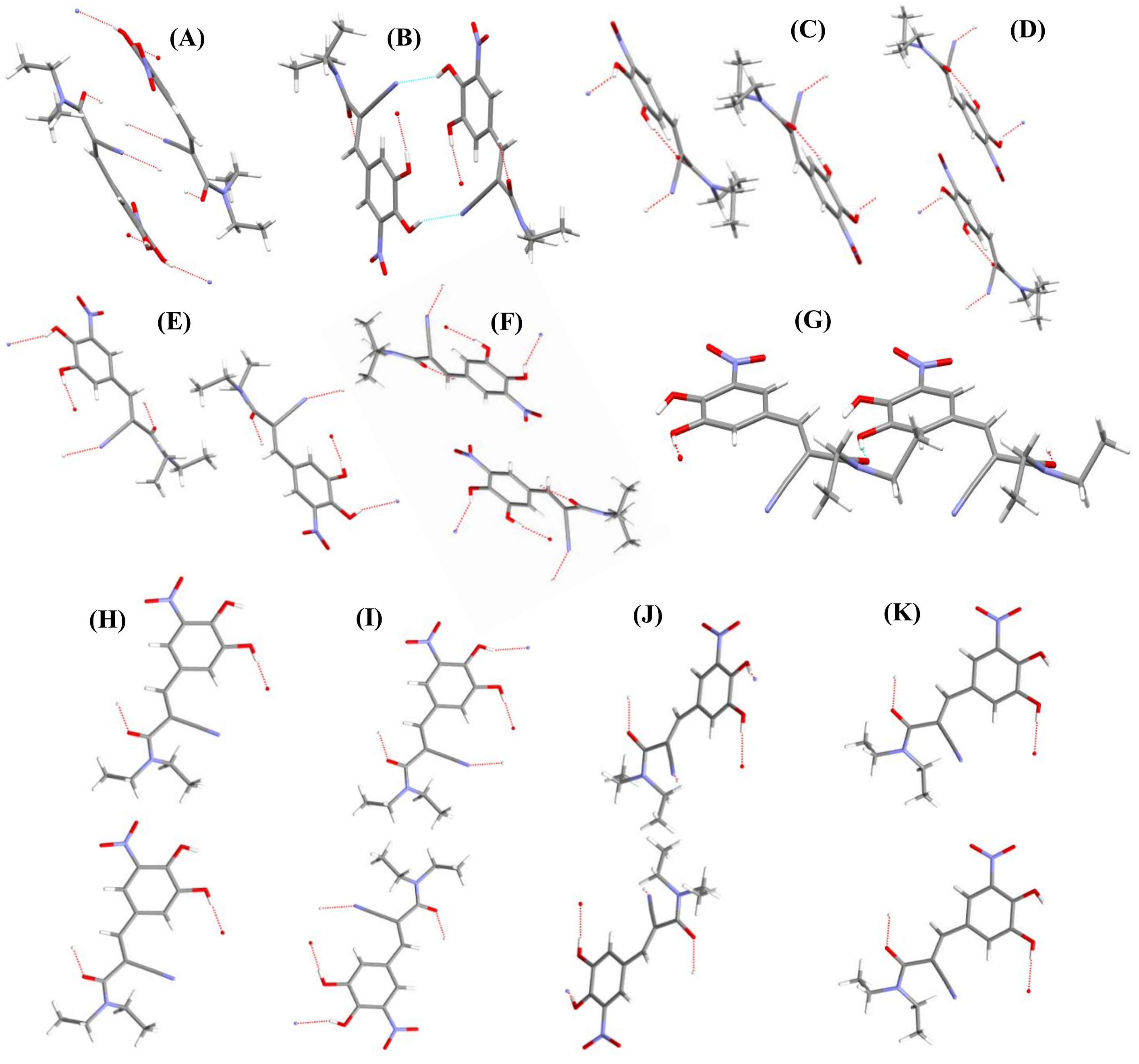

(a)

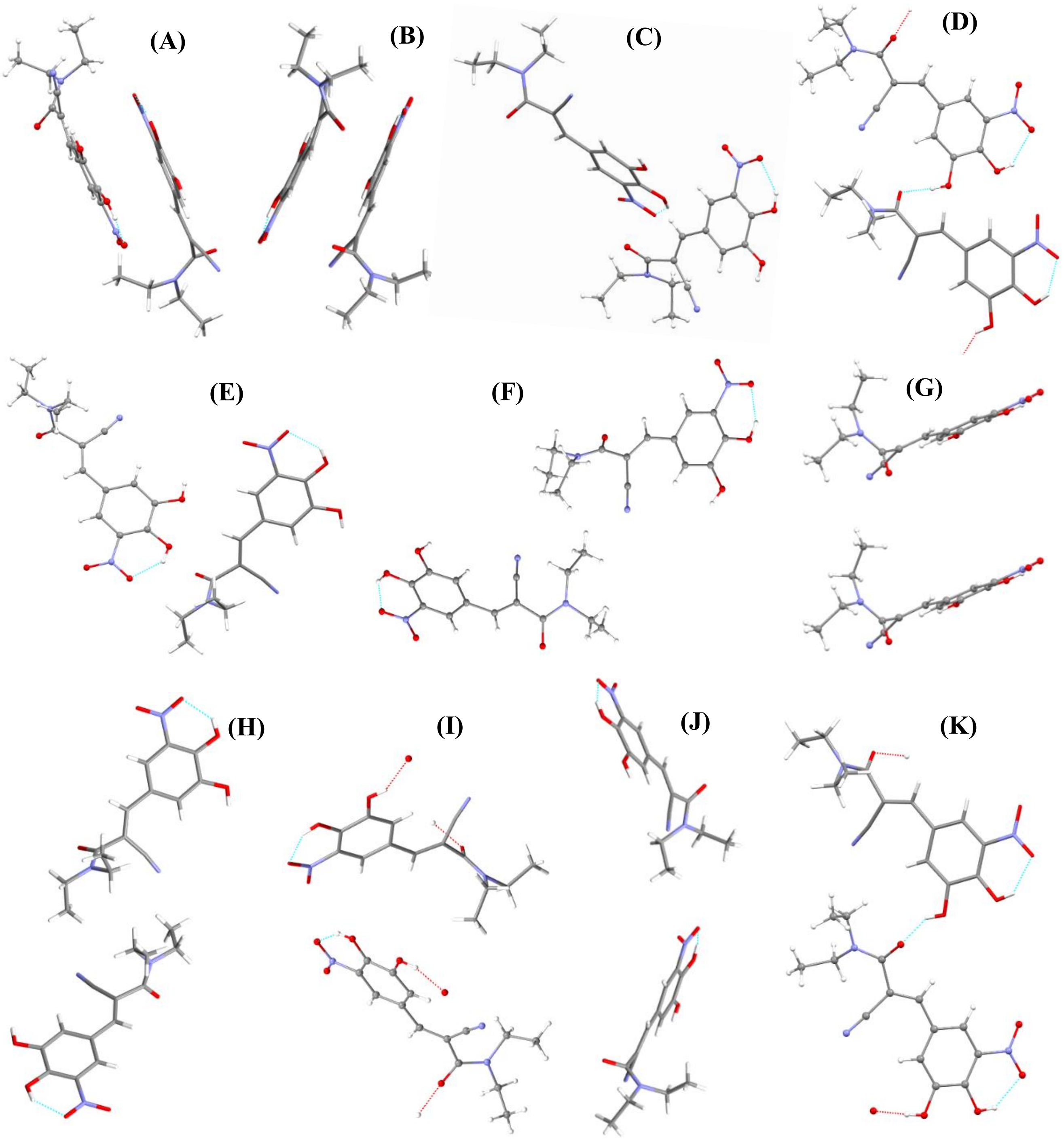

(b)

Figure S8 Energetically top-ranked pairwise intrinsic synthons (A – K) identified from the bulk structure of entacapone (a) form A and (b) form D (1st molecule (stick); 2nd molecule (ball and stick)).

Table S7. Intrinsic contributions of synthons of entacapone Form A.

Synthon	Multiplicity	Distance (Å)	vdW energy (kcal/mol)	H-bond energy (kcal/mol)	Electrostatic energy (kcal/mol)	Total energy (kcal/mol)	Main interaction
**A (221)**	1	3.87	-6.98	-	-1.0	-7.98	$\pi - \pi$
**B (166)**	1	7.61	-2.44	-0.76	-0.8	-4.00	H-bond
**C (230)**	1	6.42	-4.11	-	0.2	-3.91	vdW
**D (164)**	1	9.56	-2.51	-	-0.7	-3.21	vdW
**E (270)**	1	9.90	-2.2	-	-0.23	-2.43	vdW
**F (219)**	1	8.56	-0.97	-	-1.44	-2.41	vdW
**G (149)**	2	7.58	-0.77	-0.79	-0.05	-1.61	H-bond
**H (46)**	2	13.25	-0.97	-	-0.45	-1.42	vdW
**I (271)**	1	13.30	-1.08	-	-0.04	-1.12	vdW
**J (231)**	1	12.05	-0.68	-	-0.1	-0.78	vdW
**K (88)**	2	12.57	-0.35	-	-0.41	-0.76	vdW

Table S8. Intrinsic contributions of synthons of entacapone Form D.

Synthon	Multiplicity	Distance (Å)	vdW energy (kcal/mol)	H-bond energy (kcal/mol)	Electrostatic energy (kcal/mol)	Total energy (kcal/mol)	Main interaction
**A (780)**	1	5.16	-6.05	-	-0.92	-6.97	$\pi - \pi$
**B (92)**	1	4.51	-5.98	-	-0.11	-6.09	$\pi - \pi$
**C (360)**	1	8.17	-2.68	-	-0.70	-3.38	vdW
**D (88)**	1	7.58	-0.52	-2.04	-0.51	-3.07	H-bond
**E (23)**	1	8.12	-2.17	-	-0.68	-2.85	vdW
**F (1055)**	1	10.92	-1.59	-	-0.02	-1.61	vdW
**G (1929)**	2	7.50	-1.20	-	-0.30	-1.50	$\pi - \pi$
**H (278)**	2	10.72	-1.62	-	-0.13	-1.49	vdW
**I (82)**	1	7.98	-1.57	-	0.51	-1.06	vdW
**J (139)**	1	12.96	-0.63	-	-0.33	-0.96	vdW
**K (176)**	2	9.02	0.55	-1.10	-0.33	-0.88	H-bond

Table S9. The relative contributions of the molecular fragments to individual synthons of entacapone Form A.

Synthon	Multiplicity	Strength (kcal/mol)	% of Lattice Energy	vdW %	H-bond %	Coul %	Aliphatic (=Alkane+Alkene)	Alkane (×2)	Alkene	Aromatic ring	Nitro	Phenol (×2)	Cyanide	Amide
**A**	1	-7.98	21.86	87.47	-	12.53	29.44%	15.62%	13.82%	31.10%	12.84%	5.28%	5.94%	15.38%
**B**	1	-4.00	10.96	61.00	19.00	20.00	-0.74%	-2.56%	1.82%	20.20%	3.02%	35.22%	35.16%	7.06%
**C**	1	-3.91	10.71	105.12	-	-5.12	27.98%	22.56%	5.42%	21.82%	5.42%	4.92%	13.54%	26.32%
**D**	1	-3.21	8.79	78.19	-	21.81	14.94%	11.34%	3.60%	37.90%	37.68%	21.84%	-6.36%	-5.96%
**E**	1	-2.43	6.66	90.53	-	9.47	49.54%	48.50%	1.04%	2.84%	7.06%	0.76%	5.02%	34.72%
**F**	1	-2.41	6.60	40.25	-	59.75	69.78%	36.12%	33.66%	34.90%	42.78%	1.74%	-11.30%	-37.88%
**G**	2	-1.61	4.41	47.83	49.07	3.11	0.14%	9.88%	-9.74%	46.10%	-1.56%	-3.35%	17.81%	40.86%
**H**	2	-1.42	3.89	68.31	-	31.69	84.92%	83.79%	1.13%	7.82%	34.01%	7.75%	-1.26%	-33.21%
**I**	1	-1.12	3.07	96.43	-	3.57	10.22%	11.02%	-0.80%	-4.44%	4.92%	-1.36%	21.22%	69.36%
**J**	1	-0.78	2.14	87.18	-	12.82	0.90%	2.06%	-1.16%	-9.44%	7.16%	-7.20%	53.72%	54.84%
**K**	2	-0.76	2.08	46.05	-	53.95	77.07%	79.46%	-2.39%	7.00%	45.65%	6.55%	-13.83%	-22.45%

Table S10. The relative contributions of the molecular fragments to individual synthons of entacapone Form D.

Synthon	Multiplicity	Strength (kcal/mol)	% of Lattice Energy	vdW %	H-bond %	Coul %	Aliphatic (=Alkane+Alkene)	Alkane (×2)	Alkene	Aromatic ring	Nitro	Phenol (×2)	Cyanide	Amide
**A**	1	-6.97	19.22	86.80	-	13.20	29.66%	24.76%	4.90%	38.05%	30.79%	9.72%	-2.48%	-5.73%
**B**	1	-6.09	16.80	98.19	-	1.81	32.0%	15.6%	16.4%	45.64%	5.6%	11.04%	6.96%	-1.26%
**C**	1	-3.38	9.32	79.29	-	20.71	24.11%	22.73%	1.38%	32.14%	21.16%	19.04%	1.54%	2.03%
**D**	1	-3.07	8.47	16.94	66.45	16.61	5.29%	-3.45%	8.74%	26.64%	-2.67%	27.79%	1.15%	41.79%
**E**	1	-2.85	7.86	76.14	-	23.86	39.87%	30.39%	9.48%	28.32%	20.22%	11.67%	2.99%	-3.05%
**F**	1	-1.61	4.44	98.76	-	1.24	21.36%	31.35%	-9.99%	56.54%	-31.24%	0.72%	27.24%	25.39%
**G**	1	-1.50	4.14	80.0	-	20.0	38.39%	17.1%	21.29%	-42.61%	11.88%	1.61%	29.66%	61.08%
**H**	1	-1.49	4.11	108.72	-	8.72	22.7%	24.67%	-1.97%	15.36%	-5.56%	3.93%	31.04%	32.56%
**I**	1	-1.06	2.92	148.11	-	-48.11	-16.3%	-27.23%	10.93%	33.34%	-20.77%	45.27%	20.83%	37.68%
**J**	1	-0.96	2.65	65.63	-	34.38	85.93%	87.19%	-1.26%	9.07%	47.14%	-1.52%	-10.57%	-30.07%
**K**	1	-0.88	2.43	-62.5	125.0	37.5	-21.62%	-36.08%	14.46%	56.73%	-6.72%	-1.59%	-1.76%	74.98%

S6. Results and Discussion: Crystal Morphology and Surface Chemistry

The results of the 3D morphological simulations together with the associated surface chemistry of the dominant crystal habit faces for the two polymorphs is given in **Figure 8**. The attachment energies together with the degree of surface synthon saturation, surface area, surface energies for both forms A and D are listed in **Tables S11** and **S12** respectively, with the synthon contribution to the morphologically important faces of forms A and D being listed in **Tables S13** and **S14**.

S6.1 Crystal morphology and surface chemistry of form A

The morphology of form A (as shown in **Figure 8**) was produced based on the attachment energy of the morphologically important faces listed in **Table S11** with the morphologically important faces being displayed: three capping faces {101}, {110} and {100}, and three side faces {001}, {010} and {011}. These habit faces are in good agreement with those previously identified of form A single crystals through morphological indexation [2, 17-19]. As shown in **Figure S8**(a) and **Table S11**, the three side faces of form A are {001}, {011} and {010} with the attachment energies of -13.63 kcal/mol, -14.21 kcal/mol and -14.57 kcal/mol, respectively, hence these are the slower crystal growth faces, whilst the other three capping faces, {101}, [110] and {100}, have slightly larger attachment energies (between -16.57 and -18.87kcal/mol). The crystal habit of form A is cubic-like because the average attachment energy of the capping faces is only about -4 kcal/mol larger than the side faces.

As shown in **Table S8**(a), the side faces {001}, {010} and {011} have the higher percentage of surface saturation levels (62.66%, 61.07% and 60.08%) and lower surface energies (20.45 mJ/mol, 25.87 mJ/mol and 42.63 mJ/mol), whilst the capping faces {101}, [110] and {100} present lower levels of satisfied interactions (54.6%, 48.49% and 48.3%) and higher surface energies (35.28 mJ/mol, 54.63 mJ/mol and 67.72 mJ/mol). The average surface saturation (50.46%) of the capping faces is only 10.81% lower than the side faces (61.27%) with the average surface energy (52.54 mJ/mol) of capping faces being about 22.89 mJ/mol higher than the side faces (29.65 mJ/mol). These suggest that there is little difference in crystal growth among the crystal faces, hence the crystal habit is close to cubic.

Figure 8 shows the surface chemistry of the three capping and three side faces of entacapone form A crystal, and their extrinsic synthons contributing to the attachment energy of the corresponding individual faces are listed in **Table S13**. The three capping faces {100}, {110} and {101}, have the attachment energy contributions from both hydrogen-bonding synthons (B and G) with the two hydrogen bonds (OH-N between the phenol and cyano functional groups) of synthon B and one hydrogen bond (OH-O between phenol and amide groups) of synthon G being exposed on the face {101} as shown in **Figure 8**. However, only one OH-N bond is available on the faces {100} and {110} in addition to the one OH-O bond from synthon G. The synthons B, D, E and G-I made their contributions to the attachment energy of face {100} with synthons B-D, G, J and K contributing to face {110}, and synthons B, E-G, J and K to face {101}. The remaining synthons made their contributions to the slice energies.

For the three side faces {001}, {010} and {011}, there is no hydrogen-bonding synthon contributing to their attachment energies (**Table S13**), and this is also confirmed by the fact that no hydrogen bond

is available on these faces (**Figure 8**). The synthons C, E, H-K and D, F, H-K contribute attachment energies to faces {010} and {001}, respectively, with the face {011} containing the contributions of slice energy from synthons C-F. The remaining synthons made their contributions to the slice energies. The π-π stacking synthon A made a slice energy contribution to all faces.

Table S11. Calculated attachment energies together with the degree of surface synthon saturation, surface energies for form A.

Crystal Face	d_{hkl} (Å)	% Surface Area	Multiplicity	E_{att} (kcal/mol)	E_{sl} (kcal/mol)	% Anisotropy Factor, ξ_{hkl}	Dispersive Surface Energy, γ (mJ/m^2)	Electrostatic Surface Energy, γ (mJ/m^2)	Total Surface Energy, γ (mJ/m^2)
**{001}**	9.57	7.884	2	13.63	22.87	0.6266	15.36	5.09	20.45
**{011}**	7.51	12.204	2	14.21	22.29	0.6107	23.86	2.00	25.87
**{010}**	9.45	15.578	2	14.57	21.93	0.6008	37.80	4.83	42.63
**{101}**	6.48	16.542	2	16.57	19.93	0.5460	23.72	11.56	35.28
**{110}**	6.25	23.394	2	18.80	17.70	0.4849	41.90	12.73	54.63
**{100}**	7.40	24.396	2	18.87	17.63	0.4830	50.60	17.12	67.72
**20**	5.38	0	2	22.95	13.55	0.3712	0.00	0.00	0.00
**Whole crystal Surface Energy, γ (mJ/m^2)**							193.26	53.33	246.58

Table S12. Calculated attachment energies together with the degree of surface synthon saturation, surface energies for form D.

Crystal Face	d_{hkl} (Å)	% Surface Area	Multiplicity	E_{att} *(kcal/mol)*	E_{sl} (kcal/mol)	% Anisotropy Factor, ξ_{hkl}	Dispersive Surface Energy, γ (mJ/m^2)	Electrostatic Surface Energy, γ (mJ/m^2)	Total Surface Energy, γ (mJ/m^2)
**{020}**	12.84	27.198	2	7.6	28.72	0.7907	23.99	1.59	25.62
**{110}**	7.40	21.6366	4	15.88	20.44	0.5628	66.86	18.55	85.41
**{011}**	6.48	51.164	4	16.46	19.86	0.5468	106.55	8.66	115.20
**{111}**	5.38	0	8	22.08	14.24	0.3921	0.00	0.00	0.00
**Whole Crystal Surface Energy, γ (mJ/m^2)**							197.4	28.8	226.24

Table S13. Synthon contribution to five morphologically important faces, {100}, {110}, {101}, {010}, {011}, {001}, of entacapone Form A (capping and side faces are separated by a green line).

Synthon	Interaction energy (kcal/mol)	Distance (Å)	Slice energy (E_{sl}) or Attachment energy (E_{att})					
			{100}	{110}	{101}	{010}	{011}	{001}
A	-7.98	3.87	E_{sl}	E_{sl}	E_{sl}	E_{sl}	E_{sl}	E_{sl}
B	-4.00	7.61	E_{att}	E_{att}	E_{att}	E_{sl}	E_{sl}	E_{sl}
C	-3.91	6.42	E_{sl}	E_{att}	E_{sl}	E_{att}	E_{att}	E_{sl}
D	-3.21	9.56	E_{att}	E_{att}	E_{sl}	E_{sl}	E_{att}	E_{att}
E	-2.43	9.90	E_{att}	E_{att}	E_{att}	E_{att}	E_{att}	E_{sl}
F	-2.41	8.56	E_{sl}	E_{sl}	E_{att}	E_{sl}	E_{att}	E_{att}
G	-1.61	7.58	E_{att}	E_{att}	E_{att}	E_{sl}	E_{sl}	E_{sl}
H	-1.42	13.25	E_{att}	E_{sl}	E_{sl}	E_{att}	E_{sl}	E_{att}
I	-1.12	13.30	E_{att}	E_{sl}	E_{sl}	E_{att}	E_{sl}	E_{att}
J	-0.78	12.05	E_{sl}	E_{att}	E_{att}	E_{att}	E_{sl}	E_{att}
K	-0.76	12.57	E_{sl}	E_{att}	E_{att}	E_{att}	E_{sl}	E_{att}

Table S14. Synthon contribution to three morphologically important faces, {011}, {100}, {020}, of entacapone Form D (capping and side faces are separated by a green line).

Synthon	Interaction energy (kcal/mol)	Distance (Å)	Slice energy (Esl) or Attachment energy (Eatt)		
			{011}	{110}	{020}
A	-6.97	5.16	E_{att}	E_{sl}	E_{sl}
B	-6.09	4.51	E_{sl}	E_{sl}	E_{sl}
C	-3.38	8.17	E_{sl}	E_{sl}	E_{sl}
D	-3.07	7.58	E_{sl}	E_{sl}	E_{sl}
E	-2.85	8.12	E_{sl}	E_{att}	E_{sl}
F	-1.61	10.92	E_{att}	E_{sl}	E_{att}
G	-1.50	7.50	E_{att}	E_{sl}	E_{sl}
H	-1.49	10.72	E_{sl}	E_{sl}	E_{att}
I	-1.06	7.98	E_{sl}	E_{sl}	E_{sl}
J	-0.96	12.96	E_{att}	E_{sl}	E_{att}
K	-0.88	9.02	E_{att}	E_{att}	E_{sl}

S6.2 Crystal morphology and surface chemistry of form D

The attachment energy of the morphologically important faces of form D are listed in **Table S12** with the corresponding crystal morphology generated plotted in **Figure S9** with two side faces {110} and {020}, and one capping face {011}. The capping face {011} (**Figure S9**) of form D has an attachment energy of -16.46 kcal/mol with a surface saturation level of 54.68% (**Table S10**), whilst the side faces

{020} and {110} have the attachment energies of -7.6 kcal/mol and -15.88 kcal/mol, respectively, with the corresponding 79.07% and 56.28% of satisfied surface interactions (**Table S12**). It is clear that face {020} is the dominant morphological face as it has the smallest attachment energy (about half that of the other faces) and the highest surface saturation level. Furthermore, the surface energy of face {020} is 115.2 mJ/mol with face {110} having 85.41 mJ/mol. The face {011} has the lowest surface energy of 25.62 mJ/mol. Therefore, face {011} will grow much faster than face {020} with the growth rate of face {110} in between, leading to a long plate-like or thin fibrous crystal shape for form D, which agrees with findings in the literature [2, 19].

As shown in **Figure S9**, three habit faces {011}, {020} and {110} exist for the form D crystal with the hydrogen-bonding synthons (D and K) making no contribution to the attachment energy of face {020}. The faces {011} and {110} have attachment energy contributions from the weaker hydrogen-bonding synthon K (-0.88 kcal/mol). The synthons (A, F, G, J, K), (E, K) and (F, H, J) make contributions to the attachment energies of faces {011}, {110} and {020}, respectively, with the remaining synthons contributing to their corresponding slice energies (**Table S14**). The hydrogen bond (O-HO between the amide functional group in the second molecule and the phenol group in the first molecule) from synthon K are exposed on the face {011} as shown in **Figure S9**. This, together with the π−π stacking synthons A and B, means the capping face {011} is the fastest growing face, which was demonstrated previously by the highest surface energy and large attachment energy (**Table S12**). With the help of the weak hydrogen-bonding synthon K, the growth of the side face {110} will be faster than the slowest growing face {020} as supported by their surface and attachment energies (**Table S12**). This leads to a plate-like shaped crystal of entacapone form D.

S7. DFT and MD Simulations

As shown in **Figure S10**, the distribution of C≡N−Au bond angles and N−Au bond lengths obtained using the CCDC's Conquest indicates that the typical Au-N bond distances are in the range of 1.82 Å and 2.35 Å with a mean value of 2.03 Å, whilst the C≡N−Au bond angles lie within the range 136.2° to 180.0° with an average value of 170.6°.

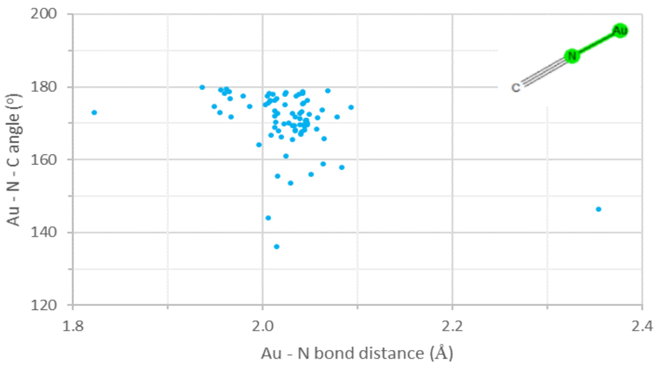

Figure S10. The CCDC Conquest search results of C≡N−Au bond angles vs N-Au bond lengths to identify the most common bond angle and length.

7.1 Atomistic modelling details

Density Functional Theory (DFT)

A face centred cubic unit cell of gold (4.08 $Å^3$) was imported into Materials Studio [21] and its lattice, atomic coordinates and electronic structure were optimised using the plane wave, pseudopotential code, CASTEP [22] within the formalism of density functional theory (DFT) [23-25]. The exchange-correlation density functional was the generalised gradient approximation of Perdew, Burke and Ernzerhof (GGA-PBE) [26], and for all systems (including those containing the entacapone molecules) the electron-ion interactions were generated on-the-fly using the PBE functional to create consistent, ultrasoft pseudopotentials [27]. The corresponding valence electron wavefunctions were expanded by a plane wave basis set corresponding to a maximum kinetic energy cut-off of 630 eV.

For optimisation of the unit cell of gold, the Brillouin zone integrations were performed on a 6 × 6 × 6 grid with 10 H symmetry-reduced k-points [28], and for the surface-plus-molecule and gas-phase systems the single sampling point corresponded to the gamma point. Following optimization of the gold unit cell we built a supercell and cleaved it to produce a 3-layer slab from which we extracted the top Au (111) surface (17.30 × 17.30 $Å^2$) to form a single layer, then extended the *c*-length to 15 Å to accommodate entacapone molecules above the surface.

Experimentally-determined crystal structures of forms A (7.58 Å × 7.69 Å × 9.91 Å) and D (15.19 Å × 25.68 Å × 7.50 Å) were used as the initial configurations of the entacapone molecules, and the lattice lengths, atomic positions and electronic structure were optimized according to the aforementioned details. Optimised molecules were then extracted from the crystal structure which formed the initial configurations for the subsequent models involving entacapone. To account for long-range dispersions we applied the pairwise, semi-empirical dispersion correction (SEDC) term of Tkatchenko and Scheffler [29] when modelling the molecule, and surface-plus-molecule systems.

For all model systems (unless stated otherwise) the geometry was optimized using the method of Broyden–Fletcher–Goldfarb–Shanno (BFGS) [30] and the self-consistent electronic minimisation method was density mixing. Further convergence details per BFGS iteration are as follows: electronic energy tolerance: 5×10^{-7} eV; energy change per ion: dE/ion 5×10^{-6} eV; maximum force: $|F|_{max}$ 0.01 eV/Å; change in displacement: $|dR|$ 5×10^{-4} Å. All calculations were non-spin polarised. The DFT calculations were run on between 8 to 384 Intel processors - E5-2650 v4.

7.2 Molecular dynamics simulations

The 3-layer gold slab constructed for the DFT models was increased along the *a*- and *b*- lengths and re-built as an orthogonal model, extending in the *x-y* plane to 75.40Å × 87.06Å and in the *z*-direction to 200Å to include a volume of vacuum above the adsorbate surface. The experimental crystal structures of the entacapone forms A and D were used as the initial molecular structures, and both the gold and entacapone structures were parametrized using the CHARMM General force field (CGenFF) [31-33]. The Au-slab was minimized then equilibrated for 5ns, and thereafter the Au-slab was fixed during all subsequent simulations unless otherwise stated.

Considering the mechanism of how the entacapone molecules might encounter the Au surface we tested three methods: layer-by-layer; Entacapone clusters encountering the surface as clumps; and Entacapone layers encountering the surface as an un-interacted tri-layer.

Method 1: Layer-by-layer adsorption

This method was tested only for form A entacapone molecules. The models consist of 50 molecules per layer, where each layer is added above the Au surface, then following minimization and equilibration the simulation continued for 100 ns. A second layer of molecules were added to the final configuration of the simulated adsorbed first layer, and the 2-layer model was then minimized and equilibrated followed by 150 ns simulation. Finally, this was repeated for the addition of the third layer for a final 150 ns simulation. Snapshots taken at stages of the simulation are shown in **Figure S11**.

Methods 2 and 3: Entacapone clusters encountering the Au surface; entacapone tri-layer encountering the Au surface

We built three layers of entacapone molecules using a total of 120 molecules, approximately 40 per layer. We estimated that this would be sufficient to allow total surface coverage and some vertical accumulation of entacapone molecules. We applied two protocols for simulating the entacapones + Au-slab:

1) The entacapone molecules were placed sufficiently far above the Au-slab such that they interacted among themselves but not with the slab, i.e. entacapone clusters would then encounter the Au surface;
2) The entacapone molecules were placed sufficiently close to the surface such that they interacted both with the slab and among themselves, i.e. an entacapone tri-layer encountered the surface.

The interactions between the slab and the entacapone molecule were depicted by the non-bonded interaction parameters taken from the work of Brandt et al [34, 35]. For simplicity no solvent was included in the models (see **Figure S12** for the initial configurations).

Following the set-up of each of the four entacapone + Au-slab systems, they were minimized for 100000 steps followed by 5ns (5000000 steps) of equilibration. In the two systems where forms A and D were sufficiently far from the Au-slab to interact among themselves only, the entacapone molecules were moved closer to the Au surface following the aforementioned minimization and equilibration steps. All four systems then underwent a NVT ensemble (i.e. a constant number of particles, temperature and volume) simulation with a time-step of 2fs at 298K for 150 ns. All simulations were performed using NAMD [36]. **Figure S12** shows snapshots of the models during the various stages of the simulations.

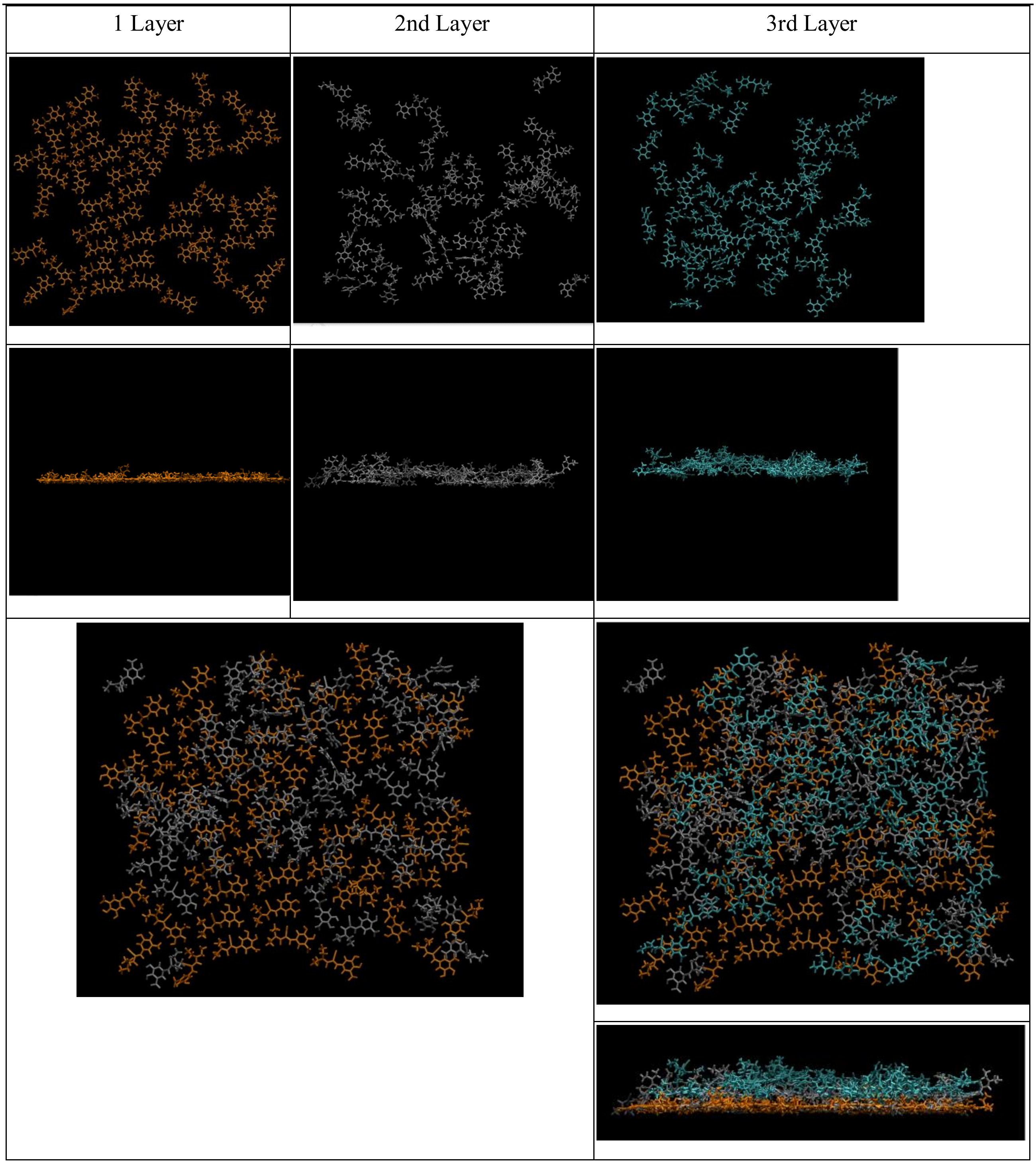

Figure S11. Final configurations: row 1 - bird's eye and row 2 - side views; row 3 – LHS bird's eye view of layers 1 and 2; RHS bird's eye and side views of all 3 layers.

**Form A entacapone interaction followed by translation to surface followed by whole system interaction. (Labelled ‘A-f’ in the main paper.)**	**Form A placed close to surface followed by whole system interaction. (Labelled ‘A-c’ in the main paper.)**	**Form D interaction followed by translation to surface followed by whole system interaction. (Labelled ‘D-f’ in the main paper.)**	**Form D placed close to surface followed by whole system interaction. (Labelled ‘D-c’ in the main paper.)**
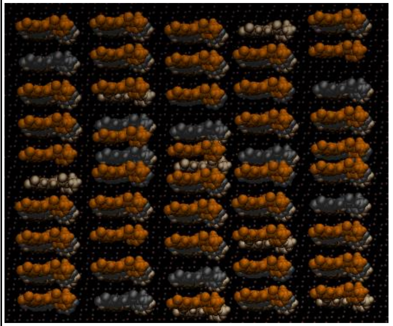 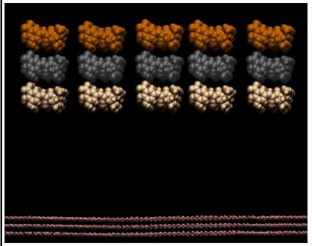	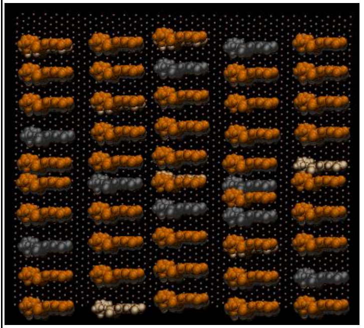 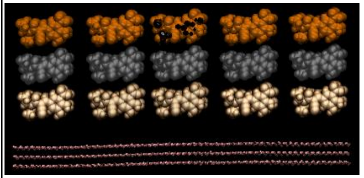	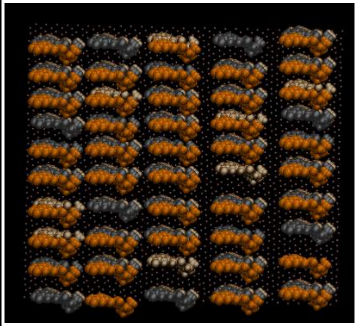 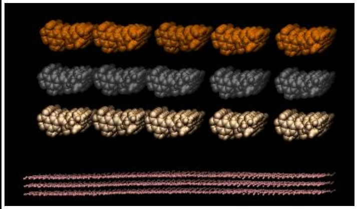	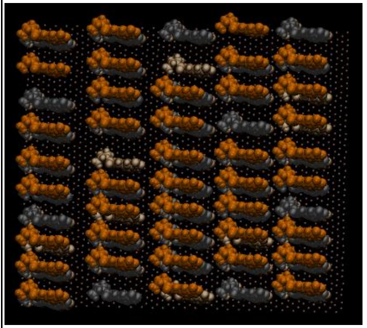 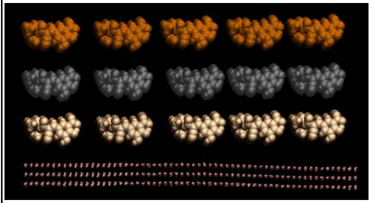
(Below: snapshot of entacapones before translation closer to the Au-slab)		(Below: snapshot of entacapones before translation closer to the Au-slab)	
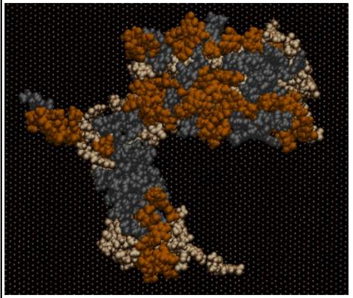		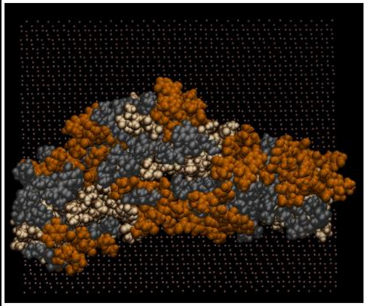	

Snapshots of final configurations following NVT simulation (from top to bottom: *x-y* plane from above the Au-slab; *x-y* plane from beneath the slab; side view)

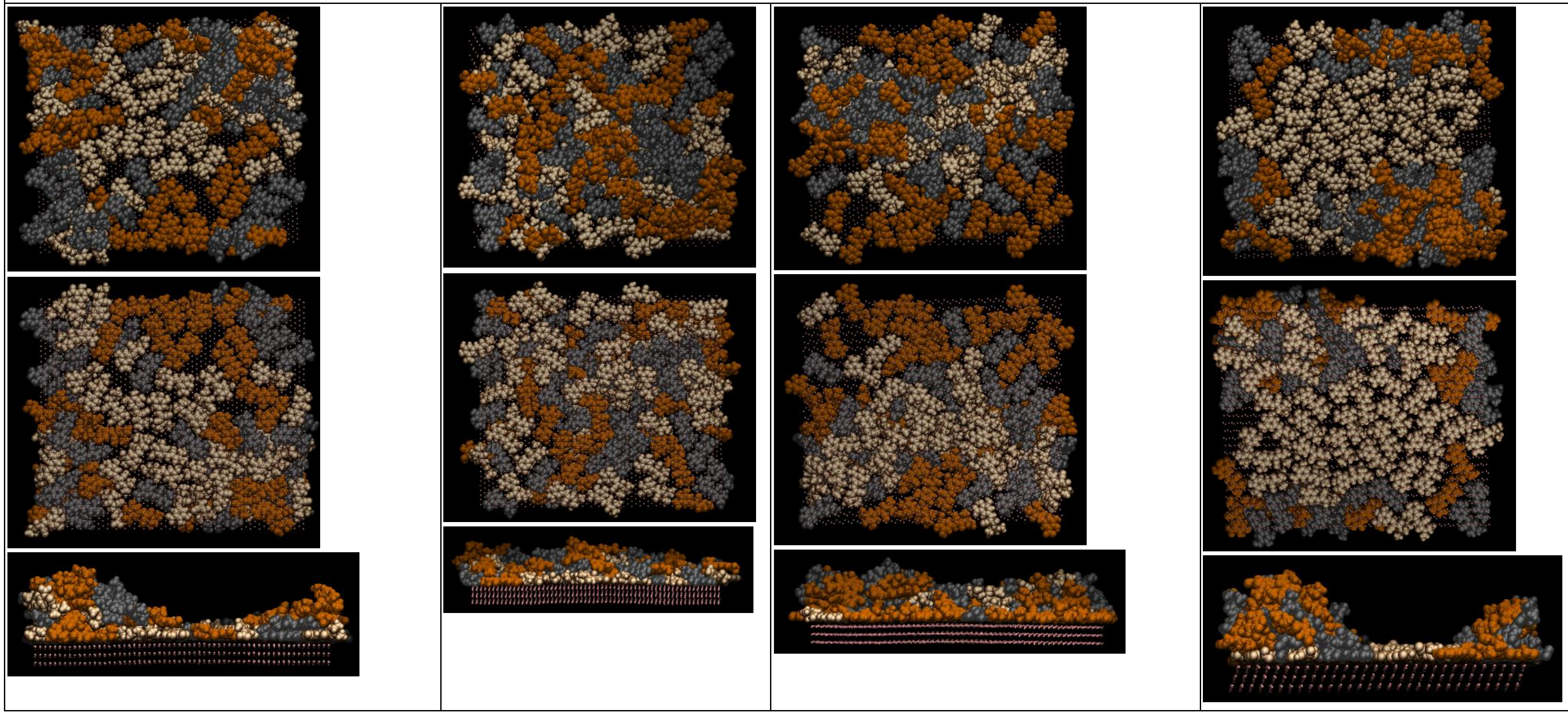

Figure S12. System configurations and snapshots both during the simulation and on its completion (bottom row).

S7.3 DFT exploration of dimers – Analogous to synthons

In crystal forms A and D neighbouring pairs of molecules were identified as interacting dimers, and these are analogous to the aforementioned synthons. For the DFT models, rather than every possible interacting pair in the bulk crystals that differ only by minor configurational changes being identified as distinct dimers, the overall general rather than specific motifs of interaction were identified as dimers. The binding energies for each of these dimers were calculated by extracting a dimer from the bulk crystal and placing it in vacuo, then allowing all atoms and electrons to optimize to their local minimum energy configuration. Twice the energy of an optimized single molecule in vacuo was subtracted from the total energy of the optimized dimer.

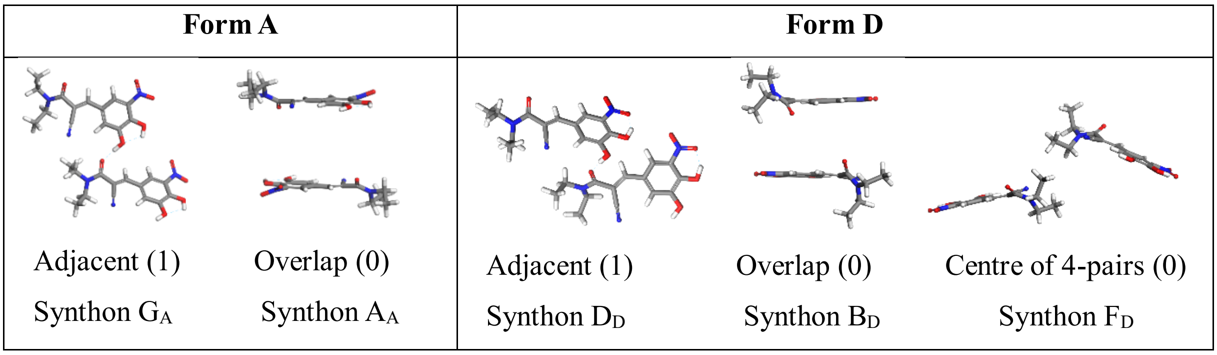

Figure S13. Overall general dimer interactions in bulk crystals with descriptive (although not literal) label (and number of intermolecular H-bonds), and also the corresponding synthons as shown in **Figure 7** and **Figure S8**.

Table S15. DFT-calculated binding energies of dimers analogous to mechanistically-identified synthons i.e. 'DFT-D_D' means a calculation of the dimer identified as synthon D of form D, and also the binding energy between forms A or D molecule and Au (Au-A or Au-D).

Dimers ('DFT-synthons')	Binding energy kcal/mol
DFT-G_A	-18.46
DFT-A_A	-6.89
DFT-D_D	-16.83
DFT-B_D	-0.22
DFT-F_D	-1.96
Au-A	-16.65
Au-D	-20.97

According to the DFT results the strongest mode of binding is found between adjacent molecules (DFT-G_A) in **Figure S13** and **Table S15**, and synthon G in **Figure S8**(a) oriented in form A, and the second strongest is found between adjacent molecules (DFT-D_D) in **Figure S13** and **Table S15**, and synthon D in **Figure 7**(b) oriented in form D.

S7.4 MD simulation results

(a)

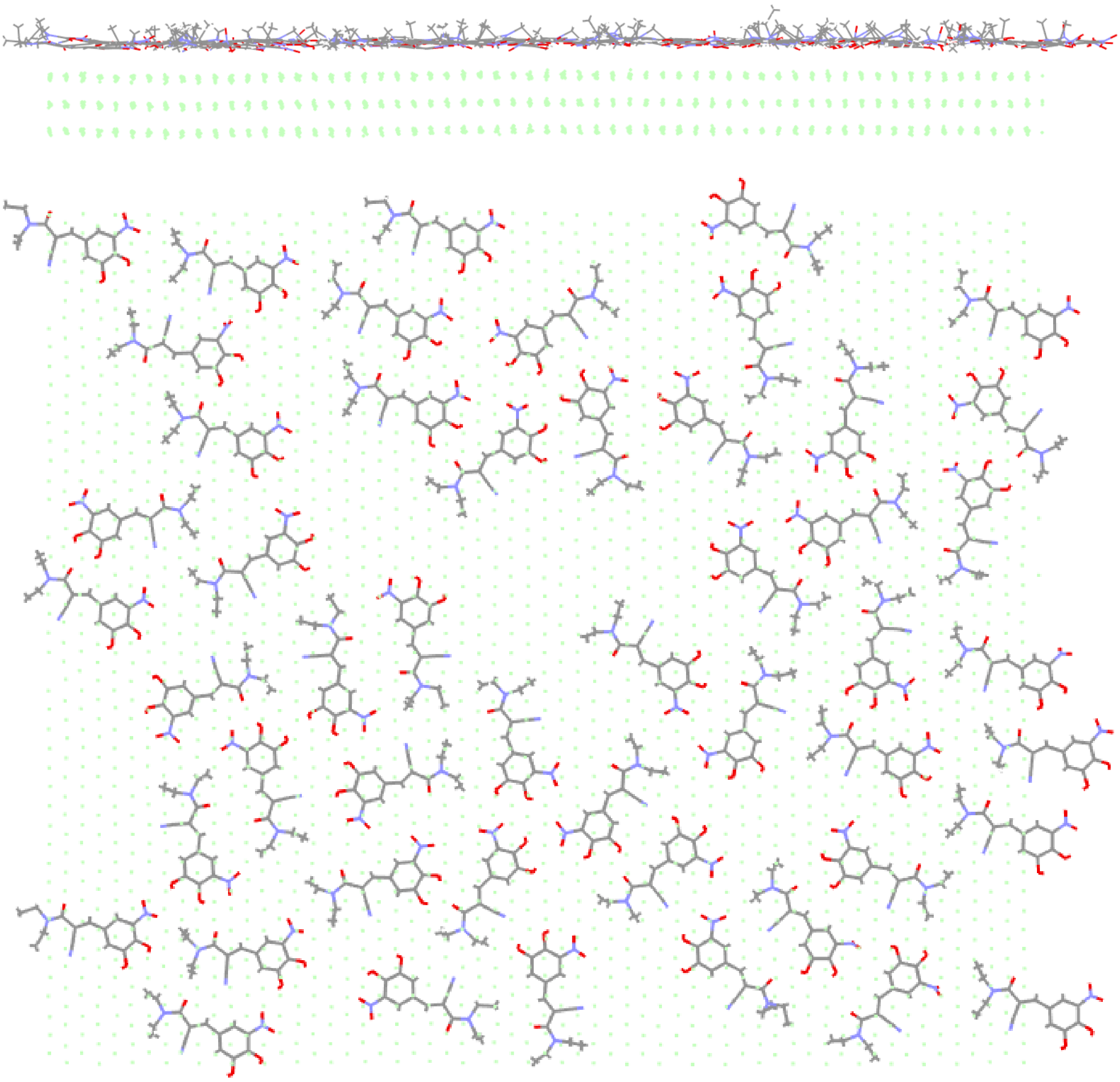

(b)

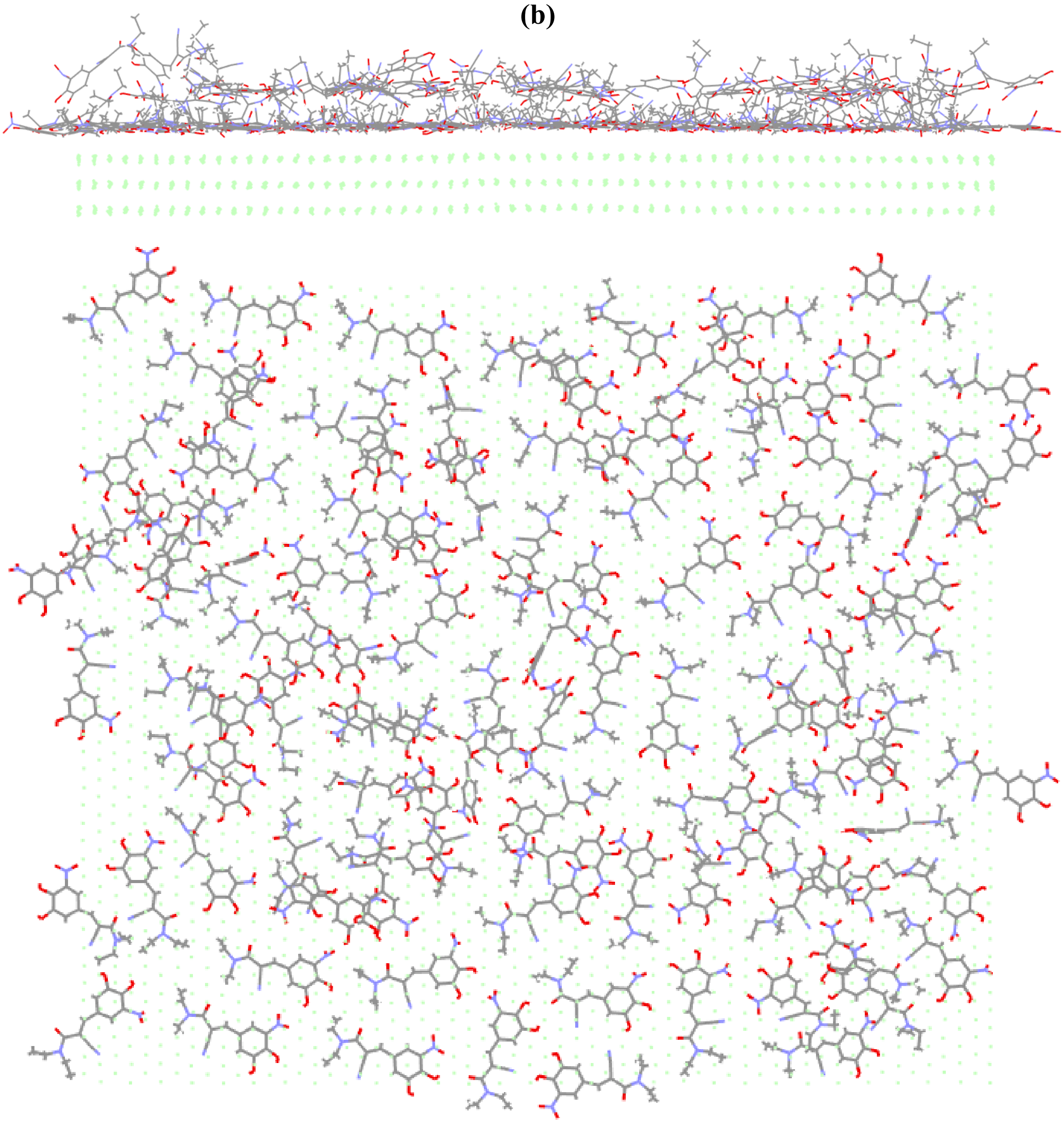

Figure S14. MD simulation results of molecular structures of the first (a) and second (b) entacapone molecule layers and Au surface.

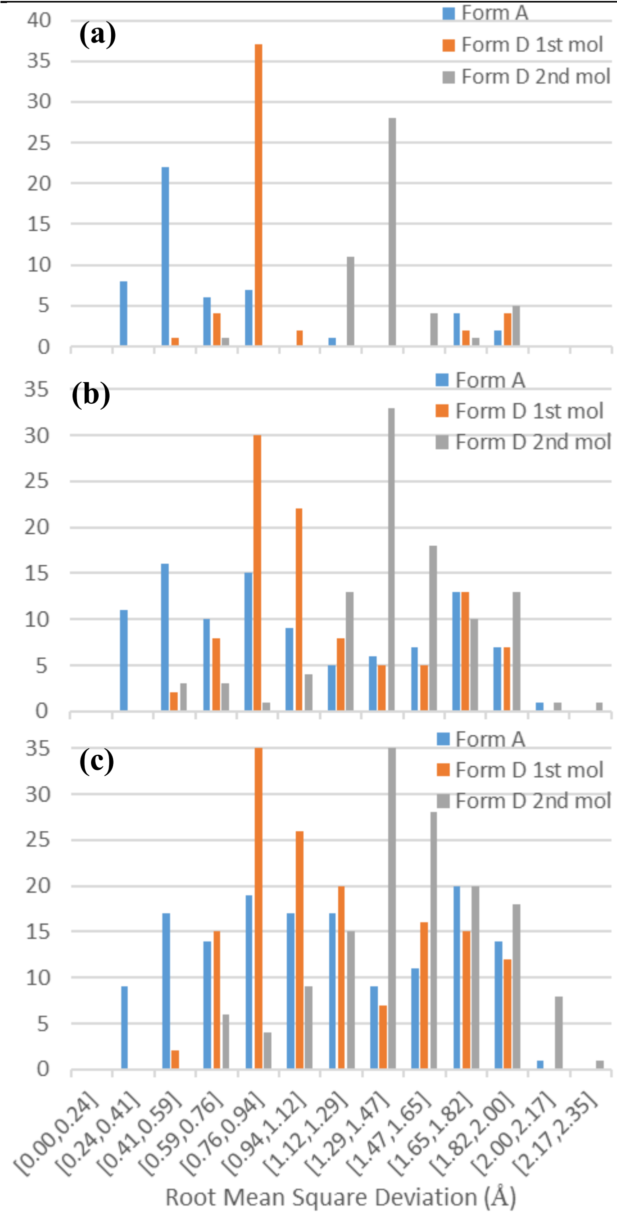

Figure S15. Molecular structure similarity: (a) one layer molecules vs Form A molecule, Form D 1st or 2nd molecule; (b) two layers molecules vs Form A molecule, Form D 1st or 2nd molecule; (c) three layers molecules vs Form A molecule, Form D 1st or 2nd molecule.

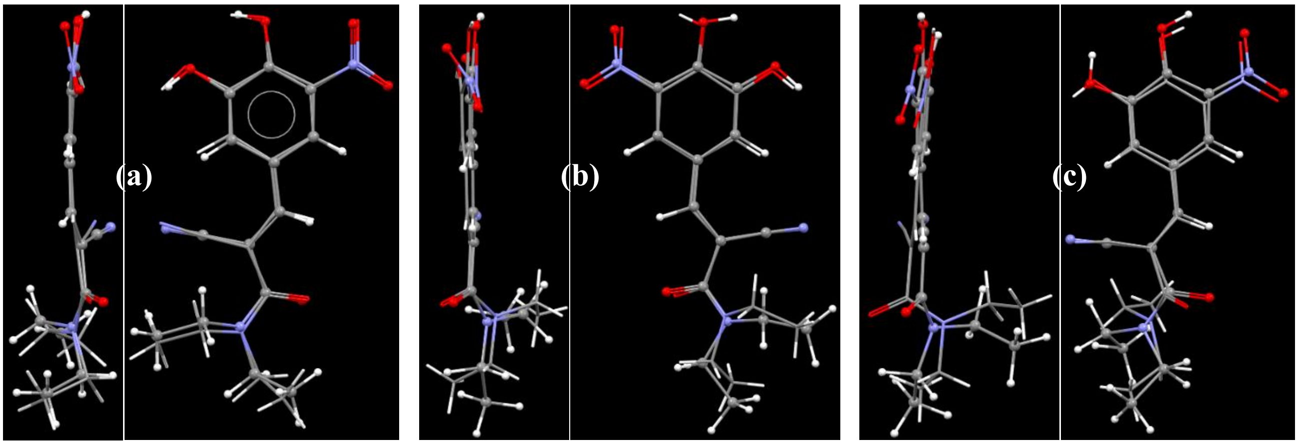

Figure S16. Overlaid molecular structures with the lowest RSMDs for (a) Form A, (b &c) Form D 1st & 2nd molecules from 1 layer MD simulations.

The interactions between entacapone molecules with the gold atoms have led to a parallel orientation of entacapone molecules (aromatic ring plane) to the surface as shown in **Figure S14**(a). The similarities between the molecules from the MD simulations (one, two and three layers) and the molecular structures from both Form A and D are plotted in **Figure S15**. The one layer MD simulations found that 60%, 82% and 78% of the MD simulated molecules are located at the root square mean deviations (RSMDs) of 0.24 – 0.59, 0.59 – 0.94 and 1.12 – 1.47 Å (corresponding to 30, 41 and 39 MD simulated entacapone molecules) for Form A, Form D 1st and 2nd molecules, respectively, as shown in **Figure S15**(a). The average numbers of molecules in each bin are about 4, 7 and 11 for Form A, Form D 1st and 2nd molecules. About 8, 1 and 1 simulated molecules were found to have the lowest RSMD ranges of 0.24 - 0.41, 0.41 – 0.59 and 0.59 – 0.76 Å. These indicate that the one layer simulated molecules have much higher similarity to Form A than Form D, hence a higher possibility to nucleate Form A on the Au surface. The overlaid molecular structures with the lowest RSMDs (0.27 for Form A, 0.47 and 0.69 for Form D 1st and 2nd molecules) from 1 layer MD simulations are plotted in **Figure S16**. For the two layers MD simulation results, the similar trend was found though the number distributions was spreaded across the RSMD range, i.e. 52%, 52% and 64% within RSMD ranges of 0.24 – 0.94, 0.76 – 1.12 and 1.12 – 1.65 Å for Form A, Form D 1st and 2nd molecules (**Figure S15**(b)) with the lowest RSMD ranges of 0.24 – 41, 0.41 – 0.59 and 0.41 – 0.59 Å having 11, 2 and 3 molecules, respectively. As shown in **Figure S15**(c), again the similar trend was found for the three layers MD simulation results with the number distributions was spreaded further across the RSMD range, i.e. 63%, 66% and 69% within RSMD ranges of 0.24 – 1.29, 0.41 – 1.29 and 1.12 – 1.82 Å for Form A, Form D 1st and 2nd molecules with the lowest RSMD ranges of 0.24 – 41, 0.41 – 0.59 and 0.59 – 0.76 Å having 9, 2 and 6 molecules, respectively.

The 10 dimers from the 1 layer MD simulation were identified using the top-ranked 10 molecules based on their similarities to Form A, Form D 1st and 2nd molecules. These dimers were then compared with the top synthons of both forms. None of the identified dimers have the close similarity of structures to the entacapone crystals. The top dimer has the potential to be similar to synthon A of crystal form A after rotating 180° and then translating in the direction perpendicular to the aromatic ring plane (**Figure S17**(a)). Similarly, the synthon E of form A crystal structure will have a very close structure to the top dimer from 2 layers MD simulation if performing an 180° rotation but no translation (**Figure S17**(b)). It was found that no synthons from Form D could potentially achieve this by simply 180° rotation, which indicates that the Form A could be nucleated on the Au surface with higher possibility.

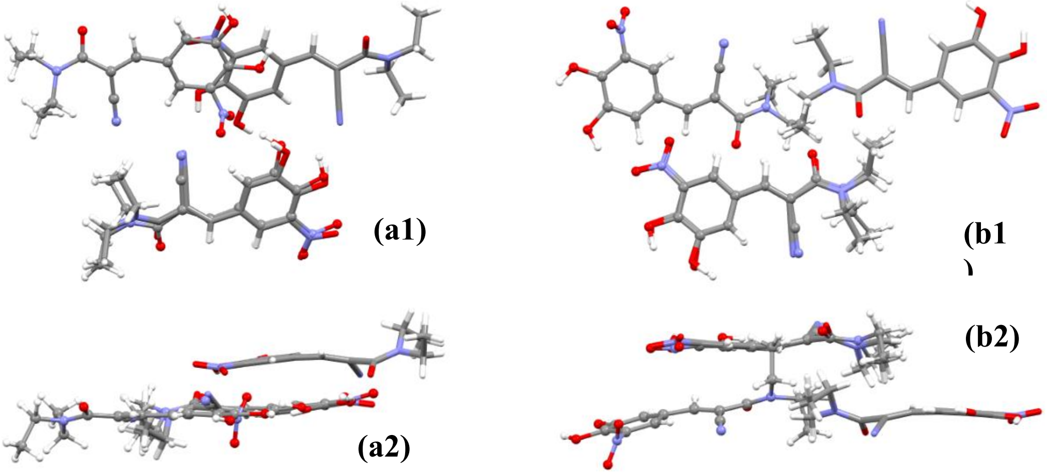

Figure S17. (a1 & a2) Synthon B of crystal Form A (stick) and top dimer (ball & stick) from 1 layer MD simulation; (b1 & b2) synthon E of crystal Form A (stick) and top dimer (ball & stick) from 2 layers MD simulation.

The similar procedure was used to analyse the MD simulation results with 3 layers of molecules. However it was found that the top dimers identified based on top molecular similarities did not produce high similarities between the corresponding top dimers and synthons of both forms. Through the scanning all top 5 dimers of all entacapone molecules against the top synthons of forms A and D, three dimers ae found to be close to synthons A and G of form A and synthon D of form D as shown in **Figure S18**. The synthon A_A and the identified MD dimer has very similar central distances between two molecules of the dimer and synthon (**Figure S18**(a2)). There is ~3 Å of displacement between the second molecules (**Figure S18**(a1)). For synthon A_G (**Figure S18**(b1&b2)), the second molecules of the dimer and synthon have rotation angles of ~5° (**Figure S18**(b1)) and ~15° (**Figure S18**(b2)). The synthon D_D as shown in **Figure S18**(c1&c2) has about 15° (**Figure S18**(c1)) and 40° (**Figure S18**(c2)) rotations between the second molecules of the dimer and synthon.

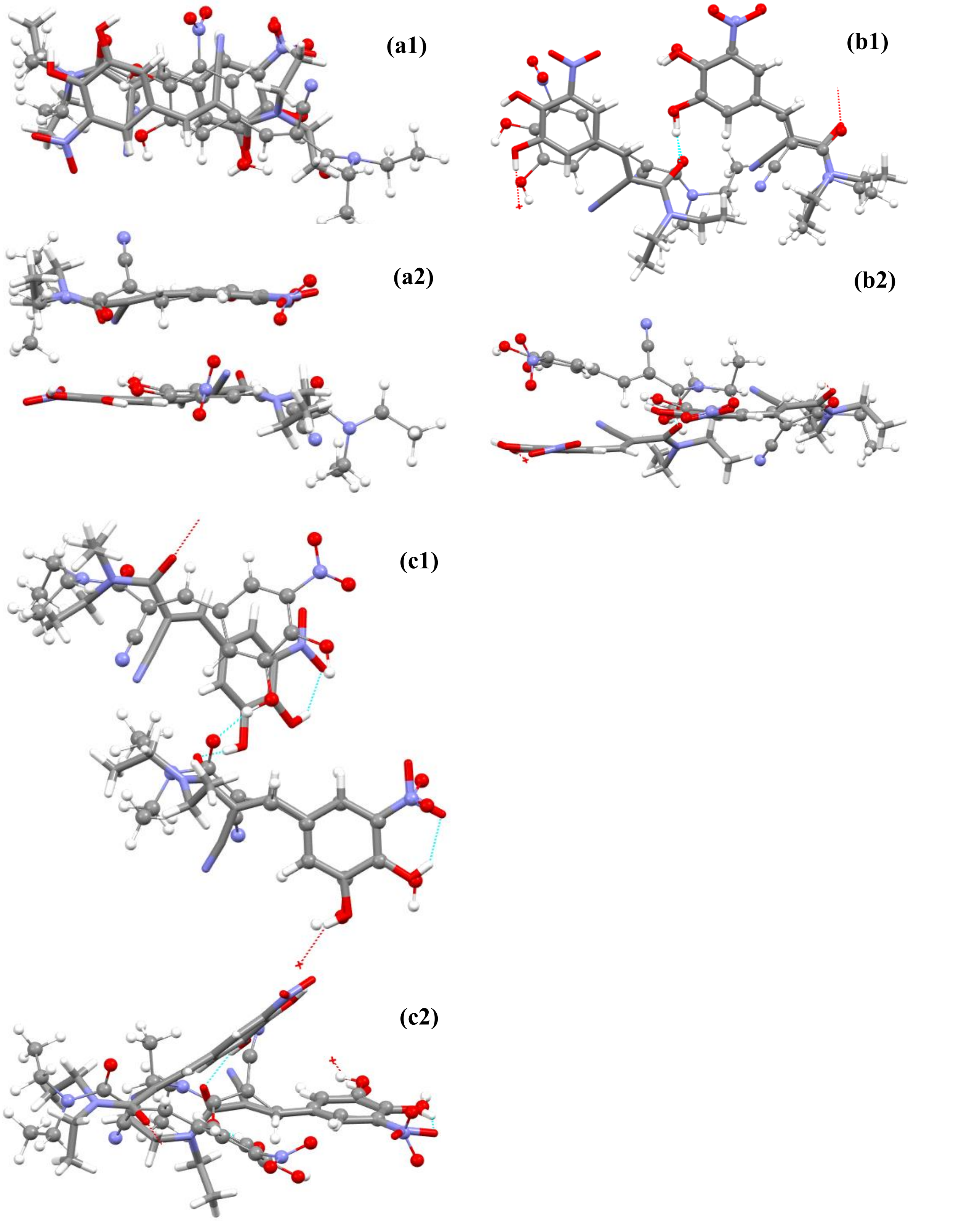

Figure S18. (a1 & a2) Synthon A of crystal Form A (stick) and one top dimer (ball & stick) from 3 layers MD simulation; (b1 & b2) synthon G of crystal Form A (stick) and one top dimer (ball & stick) from 3 layers MD simulation; (c1 & c2) synthon D of crystal Form D (stick) and one top dimer (ball & stick) from 3 layers MD simulation.

Intermolecular synthon analysis identified the top synthons for form A and D crystals (**Figure S8**). A few representative synthons (**Figure S13**) were used for DFT simulations. The binding energies of interacting molecular dimers from form D are much larger than form A for the adjacent and overlapping types. This could contribute to the formation of form D in the absence of a catalytic Au surface, i.e. in a bulk solution. However, the binding energy of form A dimer to Au surface is larger than the form D dimer (**Table 4**), indicating the establishment of stronger interactions between form A dimer (synthon G_A) and Au surface, hence leading to higher possibility of form A molecules absorbing on the Au surface. Through MD simulations with one layer entacapone molecules on the Au surface, it was found that 60% of the MD simulated molecular structures have RMSD (root mean square deviation) values in a range of 0.24 – 0.59 Å when overlapping with form A molecular structure. However, about 82% and 78% of MD simulated structures lie in much higher RMSD ranges of 0.59 – 0.94 and 1.12 – 1.47 Å for the 1st and 2nd molecular structures of form D, respectively. This indicates that the simulated structures are much more similar to form A molecular structure as there does not exist any molecules from MD simulations in the RMSD range of 0.24 – 0.59 Å when overlapping with form D molecules. Similar trend was found for the MD results with two and three layers of entacapone molecules. This further supports the finding from DFT calculations, i.e. form A molecule being the preferring one to absorb on the Au surface.

The detailed examination of the molecular and dimer structures from one, two and three layers MD simulations found that the molecules of the first layer are almost orientated parallel (referring to the aromatic ring plane) to the Au surface with higher similarity to the form A molecular structure. As a result, the one layer MD simulations did not produce any dimers similar to the top synthons of form A or form D as the molecules in the first layer have nearly identical normal distances to the Au surface (**Figure S17**(a)). With entacapone molecule-molecule interactions overtaking the interactions of molecule-Au, the molecules in the second and third layers start to show the possibility of forming synthon-like dimers. For example, the dimer identified from 2 layers MD simulations (**Figure S17**(b)) can represent synthon E_A after simple rotating. The 3 layers MD simulations produced a dimer (**Figure S18**(a)) similar to synthon A_A with only ~3 Å difference in one translation and also the synthon G_A could be reproduced after two small rotations (about 5° and 15°) of an identified dimer (**Figure S18**(b)). The closest dimer structure to a synthon of form D is shown in **Figure S18**(c). However it requires rotations of at least 15° and 40° angles. Therefore the probability of generating entacapone molecular dimers similar to the synthons from form A seems higher than form D with the trend being more obvious with the increase of molecular layers.

Overall, it may be reasonable to expect that more dimers can be identified with higher similarities to synthon structures, in particular form A synthons, as the binding energies of form A synthons to Au surface are larger than form D, the MD simulated molecules and form A molecule have higher similarity and the dimers identified from MD simulations have closer structures to form A synthons. This may be also attributed to the two molecules of form A synthons can be reproduced to each other by simple translation/slide (**Figure S8**(a)), whilst the structures of form D synthons require the

combinations of rotation and translation/slide (**Figure S8(b)**). Therefore, the dominant formation of form A molecular structure in the absorbing first layer to the Au surface produced the foundation of nucleating form A on the Au template. By considering the nucleation in the bulk solution, the molecular clusters, then nuclei, with synthons having random rotation molecules (referring to form D) may have lower energy barrier than form A synthons requiring parallel (referring to the aromatic ring plane) structures of two molecules, hence leading to the nucleation and crystallisation of form D in the bulk, i.e. without Au template.

S8. Parameters Calculated

The definitions and purposes of the parameters calculated in this study is listed in **Table 16**.

Table S16. Definitions and purposes of the parameters calculated in this study.

Calculated parameter	Definition	Purpose
a, b, c<br>*α, β, γ*	Crystal unit cell parameters	Defining the unit cell of a crystal for molecular modelling, DFT and MD
Z / Z'	Crystal structural parameters	Relating to each other through symmetry operators
q	Atomic charge	Demonstrating polarizability and for calculation of electrostatic interactions
DN···AC, DN-H<br>DN-H···AC, H···AC	Bond length and angle	Hydrogen bond network analysis for crystal and surface chemistry
RMSD	Root mean square deviation	Quality of a distribution
Molecular weight, volume, surface area<br>Crystal cell volume, packing coefficient, void space, density	Molecular and crystal properties	For molecular modelling, DFT and MD simulations and analysis
Torsion angle	Angle involving 3 adjacent linked bonds of 4 atoms in a molecule	For molecular conformation analysis
Lattice energy, functional group contribution	Crystal intrinsic and extrinsic energy, molecular fragment contributed to lattice energy	Crystal structural analysis
Synthon	Unique dimer in crystal structure	For understanding of crystal nucleation, growth, morphology and surface chemistry
Attachment energy and slice energy	Extrinsic synthon and growth layer contributions to lattice energy	For crystal growth and surface stability analysis
Surface saturation (surface anisotropy factor)	Surface property	Surface chemistry analysis
Binding energy (C≡N…Au)	Entacapone synthons and conformers binding on Au surface (Binding distance and angle)	For interaction strength analysis
Energies from vdW, hydrogen bond and coulombic interactions	Three components of lattice energy	For vdW, hydrogen bonding and electrostatic contributions

References

(1) Kwokal, A. Tailor-made Batch Crystallization of Entacapone through the Use of Self-assembled Layers on Gold Surfaces. PhD Thesis, University of Zagreb, Croatia, 2011.
(2) Bommaka, M. K.; Mannava, M. K. C.; Rai, S. K.; Suresh, K.; Nangia, A. K., Entacapone Polymorphs: Crystal Structures, Dissolution, Permeability, and Stability. *Crystal Growth & Design* **2021,** 21, 5573-5585.
(3) Clydesdale, G.; Docherty, R.; Roberts, K., HABIT95 - a program for predicting the morphology of molecular crystals as a function of the growth environment. *J. Cryst. Growth* **1996,** 166, 78-83.
(4) Clydesdale, G.; Docherty, R.; Roberts, K. J., HABIT - a program for predicting the morphology of molecular crystals. *Comput Phys Commun* **1991,** 64, (2), 311-328.
(5) Mayo, S. L.; Olafson, B. D.; Goddard, W. A., Dreiding - A Generic Force-Field For Molecular Simulations. *J Phys Chem* **1990,** 94, (26), 8897-8909.
(6) Stewart, J., MOPAC 6.0,(CQCPE program# 455). Quantum Chemistry Program Exchange, Creative Arts Building 181, Indiana University, Bloomington, IN 47405 USA. In ed.
(7) Hammond, R. B.; Hashim, R. S.; Ma, C. Y.; Roberts, K. J., Grid-based molecular modeling for pharmaceutical salt screening: Case example of 3,4,6,7,8,9-hexahydro-2H-pyrimido (1,2-a) pyrimidinium acetate. *Journal of Pharmaceutical Sciences* **2006,** 95, (11), 2361-2372.
(8) Hammond, R. B.; Ma, C. Y.; Roberts, K. J.; Ghi, P. Y.; Harris, R. K., Application of Systematic Search Methods to Studies of the Structures of Urea−Dihydroxy Benzene Cocrystals. *The Journal of Physical Chemistry B* **2003,** 107, (42), 11820-11826.
(9) Hammond, R. B.; Pencheva, K.; Ramachandran, V.; Roberts, K. J., Application of grid-based molecular methods for modeling solvent-dependent crystal growth morphology: Aspirin crystallized from aqueous ethanolic solution. *Cryst Grow Des* **2007,** 7, (9), 1571-1574.
(10) Hammond, R. B.; Pencheva, K.; Roberts, K. J., A Structural−Kinetic Approach to Model Face-Specific Solution/Crystal Surface Energy Associated with the Crystallization of Acetyl Salicylic Acid from Supersaturated Aqueous/Ethanol Solution. *Cryst Grow Des* **2006,** 6, (6), 1324-1334.
(11) Bravais, A., *Etudes Crystallographiques*. ed.; Gauthiers Villars: Paris, 1886.
(12) Donnay, J. D. H.; Harker, D., A new law of crystal morphology extending the law of bravais. *Am Mineral* **1937,** 22, (5), 446-467.
(13) Friedel, G., *Bulletin De La Societe Francaise De Mineralogie Et De Crystallographie* **1907,** 30, 326.
(14) Hartman, P.; Perdok, W. G., On the relations between structure and morphology of crystals. I. *Acta Crystallogr* **1955,** 8, (1), 49-52.
(15) Berkovitch-Yellin, Z., Toward an ab initio derivation of crystal morphology. *Journal of the American Chemical Society* **1985,** 107, (26), 8239-8253.
(16) Wulff, G.-C., Xxv. *zur frage der geschwindigkeit des wachsthums und der auflösung der krystallflächen* **1901,** 34, (1-6), 449-530.
(17) Kwokal, A.; Čavužić, D.; Roberts, K. J., Surface Adsorbed Templates for Directing the Crystal Growth of Entacapone as Monitored Using Process Analytical Techniques. *Crystal Growth & Design* **2013,** 13, 5324–5334.
(18) Kwokal, A.; Nguyen, T. T. H.; Roberts, K. J., Polymorph-Directing Seeding of Entacapone Crystallization in Aqueous/Acetone Solution Using a Self-Assembled Molecular Layer on Au (100). *Crystal Growth & Design* **2009,** 4, 4324–4334.
(19) Kwokal, A.; Roberts, K. J., Direction of the polymorphic form of entacapone using an electrochemical tuneable surface template. *CrystEngComm* **2014,** 16, 3487–3493.
(20) Berkovitch-Yellin, Z.; Van Mil, J.; Addadi, L.; Idelson, M.; Lahav, M.; Leiserowitz, L., Crystal morphology engineering by "tailor-made" inhibitors; a new probe to fine intermolecular interactions. *J Am Chem Soc* **1985,** 107, (11), 3111-3122.
(21) Dassault Systèmes, BIOVIA Materials Studio 2019 (https://www.3ds.com/products-services/biovia/products/molecular-modeling-simulation/biovia-materials-studio/), 78946 Vélizy-Villacoublay Cedex, France. In ed.; 2019.
(22) Clark, S. J.; Segall, M. D.; Pickard, C. J.; Hasnip, P. J.; Probert, M. I. J.; Refson, K.; Payne, M. C., First principles methods using CASTEP. *Zeitschrift für Kristallographie* **2005,** 220, 567-570.
(23) Hohenberg, P.; Kohn, W., Inhomogeneous Electron Gas. *Phys. Rev.* **1964,** 136, B864-B871.
(24) Kohn, W.; Sham, L. J., Self-Consistent Equations Including Exchange and Correlation Effects. *Phys. Rev.* **1965,** 140, A1133-A1138.
(25) Payne, M. C.; Teter, M. P.; Allan, D. C.; Arias, T. A.; Joannopoulos, J. D., Iterative minimization techniques for ab initio total-energy calculations: molecular dynamics and conjugate gradients. *Rev. Mod. Phys.* **1992,** 64, (4), 1045-1097.
(26) Perdew, J. P.; Burke, K.; Ernzerhof, M., Generalized Gradient Approximation Made Simple. *Phys. Rev. Lett.* **1996,** 77, (18), 3865-3868.
(27) Vanderbilt, D., Soft self-consistent pseudopotentials in a generalized eigenvalue formalism. *Phys. Rev. B* **1990,** 41,

(11), 7892-7895.
(28) Monkhorst, H. J.; Pack, J. D., Special points for Brillouin-zone integrations. *Phys. Rev. B* **1976,** 13, (12), 5188-5192.
(29) Tkatchenko, A.; Scheffler, M., Accurate Molecular Van Der Waals Interactions from Ground-State Electron Density and Free-Atom Reference Data. *Phys. Rev. Lett.* **2009,** 102, 073005.
(30) Pfrommer , B. G.; Coté , M.; Louie, S. G.; Cohen, M. L., Relaxation of Crystals with the Quasi-Newton Method. *J. Comp. Phys.* **1997,** 131, (1), 233-240.
(31) Vanommeslaeghe, K.; Hatcher, E.; Acharya, C.; Kundu, S.; Zhong, S.; Shim, J.; Darian, E.; Guvench, O.; Lopes, P.; Vorobyov, I.; Mackerell Jr, A. D., CHARMM general force field: A force field for drug-like molecules compatible with the CHARMM all-atom additive biological force fields. *Journal of Computational Chemistry* **2010,** 31, (4), 671-690.
(32) Vanommeslaeghe, K.; Mackerell Jr, A. D., Automation of the CHARMM General Force Field (CGenFF) I: Bond perception and atom typing. *Journal of Computational Chemistry* **2012,** 52, (12), 3144-3154.
(33) Vanommeslaeghe, K.; Raman, E. P.; Mackerell Jr, A. D., Automation of the CHARMM General Force Field (CGenFF) II: Assignment of bonded parameters and partial atomic charges. *Journal of Computational Chemistry* **2012,** 52, (12), 3155-3168.
(34) Brandt, E. G.; Lyubartsev, A. J., Systematic Optimization of a Force Field for Classical Simulations of TiO2–Water Interfaces. *J. Phys. Chem. C* **2015,** 119, (32), 18110–18125.
(35) Brandt, E. G.; Lyubartsev, A. J., Molecular Dynamics Simulations of Adsorption of Amino Acid Side Chain Analogues and a Titanium Binding Peptide on the TiO2 (100) Surface. *J. Phys. Chem. C* **2015,** 119, (32), 18126–18139.
(36) Phillips, J. C.; Hardy, D. J.; Maia, J. D. C.; Stone, J. E.; Ribeiro, J. V.; Bernardi, R. C.; Buch, R.; Fiorin, G.; Henin, J.; Jiang, W.; McGreevy, R.; Melo, M. C. R.; Radak, B. K.; Skeel, R. D.; Singharoy, A.; Wang, Y.; Roux, B.; Aksimentiev, A.; Luthey-Schulten, Z.; Kale, L. V.; Schulten, K.; Chipot, C.; Tajkhorshid, E., Scalable molecular dynamics on CPU and GPU architectures with NAMD. *Journal of Chemical Physics* **2020,** 153, 044130.
